# Design of a multi-epitope vaccine against brucellosis fused to IgG-fc by an immunoinformatics approach

**DOI:** 10.3389/fvets.2023.1238634

**Published:** 2023-10-23

**Authors:** Aodi Wu, Yueli Wang, Adnan Ali, Zhenyu Xu, Dongsheng Zhang, Kairat Zhumanov, Jinliang Sheng, Jihai Yi

**Affiliations:** ^1^College of Animal Science and Technology, Shihezi University, Shihezi, Xinjiang, China; ^2^College of Veterinary Medicine, Kazakhstan Kazakh State Agricultural University, Almaty, Kazakhstan

**Keywords:** *Brucella*, vaccine, epitope, immunoinformatics, IgG-fc

## Abstract

**Introduction:**

*Brucella*, a type of intracellular Gram-negative bacterium, has unique features and acts as a zoonotic pathogen. It can lead to abortion and infertility in animals. Eliminating brucellosis becomes very challenging once it spreads among both humans and animals, putting a heavy burden on livestock and people worldwide. Given the increasing spread of brucellosis, it is crucial to develop improved vaccines for susceptible animals to reduce the disease’s impact.

**Methods:**

In this study, we effectively used an immunoinformatics approach with advanced computer software to carefully identify and analyze important antigenic parts of *Brucella abortus*. Subsequently, we skillfully designed chimeric peptides to enhance the vaccine’s strength and effectiveness. We used computer programs to find four important parts of the *Brucella* bacteria that our immune system recognizes. Then, we carefully looked for eight parts that are recognized by a type of white blood cell called cytotoxic T cells, six parts recognized by T helper cells, and four parts recognized by B cells. We connected these parts together using a special link, creating a strong new vaccine. To make the vaccine even better, we added some extra parts called molecular adjuvants. These included something called human β-defensins 3 (hBD-3) that we found in a database, and another part that helps the immune system called PADRE. We attached these extra parts to the beginning of the vaccine. In a new and clever way, we made the vaccine even stronger by attaching a part from a mouse’s immune system to the end of it. This created a new kind of vaccine called MEV-Fc. We used advanced computer methods to study how well the MEV-Fc vaccine interacts with certain receptors in the body (TLR-2 and TLR-4).

**Results:**

In the end, Immunosimulation predictions showed that the MEV-Fc vaccine can make the immune system respond strongly, both in terms of cells and antibodies.

**Discussion:**

In summary, our results provide novel insights for the development of *Brucella* vaccines. Although further laboratory experiments are required to assess its protective effect.

## 1. Introduction

*Brucella* is a Gram-negative bacterium that lives inside cells and can cause reproductive problems in animals and chronic illnesses in humans (1). This disease, known as brucellosis or by other names like Wave fever or Malta fever, is a widespread bacterial infection affecting both animals and humans in various regions around the world. It is considered one of the most common bacterial zoonotic diseases globally (2). *Brucella* has a strong ability to invade a specific type of white blood cell called macrophages, which makes it resistant to many antibiotics commonly used against other bacteria. As a result, this harmful pathogen poses a significant threat to global public health, causing considerable social and economic challenges (3).

Controlling brucellosis in livestock currently relies on using *Brucella abortus* S19, *B. abortus* RB51, and *B. melitensis* Rev-1 strains (4). However, completely removing the remaining virulence associated with these weakened vaccine strains is a challenging task. There is also a risk that these vaccine strains could infect humans, which might worsen the spread of the disease. Furthermore, these strains can induce abortions in pregnant animals, causing significant economic losses. Additionally, their presence complicates the accurate diagnosis and management of brucellosis (5, 6). Consequently, we are facing significant challenges when it comes to diagnosing and treating brucellosis, emphasizing the urgent need for a safe and effective vaccine or therapy (7).

In light of these challenges, peptide vaccines offer a promising approach to combat brucellosis. Not only do they stimulate strong antibody responses, but they also address the safety concerns associated with live vaccines (7). Peptide-based interventions provide a safer and practical alternative for dealing with the devastating impact of brucellosis.

In previous studies, researchers have explored a range of outer membrane proteins (OMPs) and effector proteins as potential immunodominant antigens against *Brucella*. Notably, two such proteins, OMP16 and OMP19, which are outer membrane lipoproteins, are prominently found on the surface of all *Brucella* strains (8). These lipoproteins are universally present in *Brucella*.OMP16, similar to a protein called peptidoglycan-associated lipoprotein (PAL) found in other Gram-negative bacteria, is highly conserved and plays a crucial role in maintaining the structural integrity and function of the outer membrane. Interestingly, OMP16 also acts as a pathogen-associated molecular pattern (PAMP) in *Brucella abortus*, which means it triggers the activation of dendritic cells (DCs) in the body, leading to a strong Th1 immune response (9). Encouragingly, recombinant OMP16 has been shown to generate a potent protective immune response in mice, making it a highly promising candidate for a vaccine (10).

In contrast, OMP19 is resistant to protease degradation, particularly following oral infection with *Brucella abortus*. Additionally, it acts as a protective shield for another protein called OMP25, preventing it from being degraded by proteases (11).

Intriguingly, It has been revealed that OMP19 inhibits MHC-II expression and hinders antigen presentation, prevents T cell recognition to evade host immunity and establishes chronic infections (12). Because of its strong protective qualities against *Brucella*, OMP19 looks like a very promising candidate for a vaccine to fight brucellosis (13).

OMP25 is an important protein on the outer membrane of *Brucella abortus*. It helps keep the cell’s outer covering strong and intact (14). Interestingly, OMP25 also plays a role in reducing the production of certain immune signals like tumor necrosis factor-alpha (TNF-α) and interleukin 12 (IL-12), which are typically produced when the body is fighting infections caused by other germs. This shows that OMP25 has a significant effect on the immune system (15). Studies have shown that vaccines made from OMP25’s DNA and protein can protect mice from *Brucella abortus* infection, making OMP25 a strong candidate for a vaccine (16, 17).

Another protein called *Brucella* ribosomal protein L7/L12 is known for being easily recognized by the immune system and staying similar across different *Brucella* types (18). This protein can activate a specific type of white blood cell called monocytes in animals with infections. This activation leads to the production of interferon-gamma, an important immune molecule that helps protect against *Brucella abortus* infection (19, 20). Because of these proteins’ ability to trigger immune responses, they seem like good options for designing vaccines that can delay or prevent brucellosis.

Antibodies are made of two parts: the antigen binding fragment (Fab) and the crystallizable fragment (Fc). Using Fc fragment fusion protein technology is a very effective way to make protein and peptide drugs last longer in the body. Important biological molecules found on the surface of cells, like cell receptors, cytokines, enzymes, and peptide antigens on harmful germs, can be combined with Fc parts. This makes these molecules more stable in the body and helps them last longer, making their effects stronger (21, 22). When we combine specific antigens with Fc parts, it creates a new and promising strategy for vaccines to fight diseases (23).

A study by David G. Alleva showed that when AKS-452 was fused with the Fc fragment, it produced much higher levels of neutralizing antibodies in mice compared to when Fc was not used. This significant boost in the body’s response highlights the enormous potential of using the immunoglobulin Fc fragment fusion in peptide vaccines (24). Using this method for making vaccines has a lot of promise for various treatments and preventive measures.

Previous studies have shown that *Brucella* multi-epitope vaccines (MEVs), created by predicting important parts recognized by the immune system, offer a level of immune protection, although not as effective as weakened vaccines. However, a new approach using peptide vaccines that include multi-epitope molecules fused to IgG-Fc for fighting *Brucella* infections has not been explored much. So, we combined the IgG Fc fragment with a multi-epitope structure, forming the peptide molecule MEV-Fc. This molecule includes important parts from different immune responses like CTL, HTL, B cells, hBD-3, PADRE, and IgG Fc.

**Figure 1:**
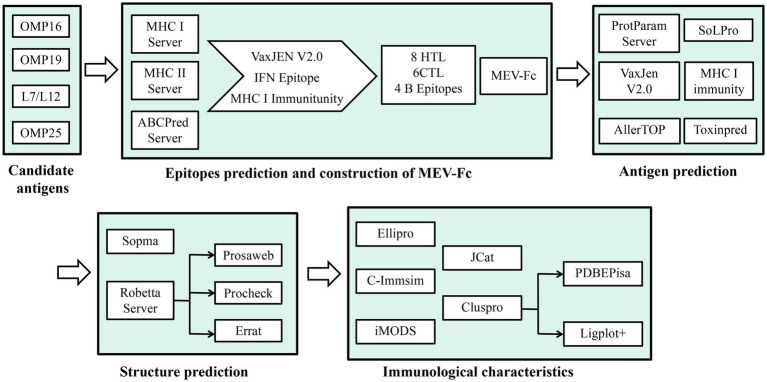
Illustrates the construction and analysis flow chart of MEV-Fc.

To understand how effective this combined molecule could be, we used predictive tools to study its features, structure, and how it interacts with immune receptors such as TLR2 and TLR4. Additionally, we used a tool called C-ImmSim server to simulate how the immune cells would respond after the molecule is given as a vaccine to mice (Figure 1). Our main goal in this study was to create a peptide molecule that could enhance the immune protection provided by *Brucella* subunit vaccines. This could be a groundbreaking approach in preventing brucellosis.

## 2. Materials and methods

### 2.1. Sequence retrieval

The amino acid sequences of the main *Brucella* antigens, specifically OMP16 (AEF59023.1), OMP19 (AAB06277.1), L7/L12 (AAL51929.1), and OMP25 (AFJ79953.1), were retrieved from the NCBI database and are detailed in Table 1. To determine whether these four proteins have antigenic properties, we employed the VaxiJen server v2.0, setting a threshold value at 0.5 (25).

**Table 1:** Protein information: L7/L12, OMP16, OMP19, OMP25.

Protein name	Sequence	Vaxijen score	Antigenicity
OMP16	MRRIQSIARSPIAIALFMSLAVAGCASKKNLPNNAGDLGLGAGAATPGSSQDFTVNVGDRIFFDLDSSLIRADAQQTLSKQAQWLQRYPQYSITIEGHADERGTREYNLALGQRRAAATRDFLASRGVPTNRMRTISYGNERPVAVCDADTCWSQNRRAVTVLNGAGR	0.5500	ANTIGEN
OMP19	MGISKASLLSLAAAGIVLAGCQSSRLGNLDNVSPPPPPAPVNAVPAGTVQKGNLDSPTQFPNAPSTDMSAQSGTQVASLPPASAPDLTPGAVAGVWNASLGGQSCKIATPQTKYGQGYRAGPLRCPGELANLASWAVNGKQLVLYDANGGTVASLYSSGQGRFDGQTTGGQAVTLSR	0.6547	ANTIGEN
OMP25	MTFKNLLGASLVAVITSTSAYAADAIVAQEPAPIAIAPSFSWAGAYFGGQVGYGWGRAKLENRTNGGTSEFKPNGFIGGLYTGYNFDTGNNFILGLDANVDYNNLKKSRDFITSGNPVQTTGETQLRWSGAVRARAGYAIDRFMPYIAGGVAFGGIKNSLRIGGEESSKSKTQTGWTVGAGIEYAATDNVLLRLEYRYTDYGKKNFGLNDLDTRGSFKTNDIRLGVAYKF	0.7575	ANTIGEN
L7/L12	MNTRASNFLAASFSTIMLVGAFSLPAFAQENQMTTQPARIAVTGEGMMTASPDMAILNLSVLRQAKTAREAMTANNEAMTKVLDAMKKAGIEDRDLQTGGINIQPIYVYPDDKNNLKEPTITGYSVSTSLTVRVRELANVGKILDESVTLGVNQGGDLNLVNDNPSAVINEARKRAVANAIAKAKTLADAAGVGLGRVVEISELSRPPMPMPIARGQFRTMLAAAPDNSVPIAAGENSYNVSVNVVFEIK	0.5381	ANTIGEN

### 2.2. Prediction of cytotoxic T-lymphocyte epitopes

To predict Cytotoxic T Lymphocyte (CTL) epitopes for the primary *Brucella* antigen, we utilized the IEDB MHC I server, accessible (26).^1^ The specific parameters we used were as follows: Prediction Method: IEDB recommended 2020.09 (NetMHCpan EL 4.1); Source species of MHC: Human; HLA allele reference set, and Length set to 9 and 10. We selected epitopes with a percentile rank of less than 0.5 for further analysis. We also used Class I Immunogenicity to predict immunogenicity and chose CTL epitopes with a percentile rank of less than 0.5 and an immunoscore greater than 0 for further analysis. To determine the antigenicity of these screened epitopes, we employed the VaxiJen server v2.0, setting a threshold at 0.5 (25). The epitopes that passed these criteria were considered as immunodominant CTL epitopes for the construction of MEV-Fc.

### 2.3. Prediction of helper T-lymphocyte epitopes

To predict Helper T Lymphocyte (HTL) epitopes for the dominant *Brucella* antigen, we utilized the IEDB MHCII server 2, which can be accessed (27).^2^ The specific parameters we used were as follows: Prediction Method: IEDB recommended 2.22; MHC source species: Human; MHC allele set selected as all reference. We set the epitope length to 15. The predicted epitopes were ranked based on percentile rank, and we selected those with a percentile rank of less than 0.5. To predict their antigenicity, we used the VaxiJen server v2.0 with a threshold set at 0.5 (25). We chose epitopes with an antigenicity value greater than 0.5, and we predicted their inducibility of IFN-γ using an epitope server (28) specifically designed for IFN-γ. Ultimately, the epitope that was predicted to induce IFN-γ was selected as the HTL immunodominant epitope for the construction of MEV-Fc.

### 2.4. Prediction of linear B-cell epitopes

To predict linear B-cell epitopes for the dominant *Brucella* antigen, we used the ABCpred server, which is accessible,^3^ with the following parameters: epitope length = 16 and screening threshold = 0.51. We employed thresholds ranging from +0.1 to +1.0 (29). The highest ABCpred score among these thresholds was selected as the immunodominant B-cell epitope for MEV (Multi-Epitope Vaccine) construction.

### 2.5. Construction of MEV-fc

To create the MEV, we used an appropriate linker to connect the individual epitopes. This linker prevents these epitopes from interacting with each other. The MEV was formed by connecting the predicted and screened CTL epitopes, an HTL epitope, and a linear B-cell epitope using the GPGPG linker.

To further enhance its ability to trigger an immune response and ensure the persistence of the helper T cell 1 (TH1) response (30), we added the hBD-3 sequence (PDB ID: 1KJ6) and the PADRE sequence to the N-terminal end of the MEV, using EAAAK linkers. Finally, to make the peptide more effective at triggering an immune response and to make it last longer in the body, we added the sequence of the mouse immunoglobulin IgG Fc fragment (P01868-1) by connecting it to the C-terminus of the MEV with KK linkers. This resulted in the complete sequence of the vaccine construct.

### 2.6. Evaluation of physical and chemical properties of vaccines

We evaluated the properties of MEV-Fc using bioinformatic analysis software. Firstly, we analyzed its physical and chemical characteristics using the ProtParam server, accessible.^4^ Next, we conducted antimicrobial and immunogenicity analyses using the VaxiJen v2.0 server. We specifically utilized the IEDB I class immunogenicity module, with VaxiJen predictions showing an accuracy range of 70 to 89% (25). Subsequently, we predicted solubility using the SOLpro server, available,^5^ which demonstrated an overall accuracy of 74.15% (31). Finally, we performed sensitivity and toxicity analyses using AlergenFP v1.0, accessible (32),^6^ and ToxinPred, available (33).^7^

### 2.7. Prediction of secondary and tertiary structures

We predicted the secondary structure of MEV-Fc using the SOPMA secondary structure prediction tool. To forecast the tertiary structure of MEV-Fc, we utilized the Robetta server (34). To assess the quality of the constructed models, we employed the Prosa Web Server (35), the Ramachandran Plot, and the PROCHECK Server (36), which can be accessed.^8^ These tools help evaluate the correctness and quality of the protein’s three-dimensional structure. We further assessed the uncertainty of the tertiary structure through ERRAT, available.^9^ ERRAT examines the overall quality factor of non-bonded atomic interactions, with higher values indicating better quality (37).

### 2.8. Prediction of B-cell epitopes

B-cell epitopes are particular sites on antigens where B-cell antibodies can bind and trigger immune responses (38). In vaccine development, these epitopes play a crucial role in generating an effective immune response. To predict B-cell epitopes for MEV-Fc, we utilized the ElliPro server with default parameters (39).

### 2.9. Molecular docking of MEV-fc and immune receptors TLR2 and TLR4

Molecular docking is a fundamental and promising method for studying how peptides interact with human immune receptors like TLR2 and TLR4 (40). It offers a quick and cost-effective way to understand the detailed interactions at the atomic level within the structure of the antibody–antigen complex and its interface.

Here’s how we conducted the molecular docking analysis: We obtained the PDB files of TLR2 (PDB ID: 2Z7X) and TLR4 (PDB ID: 2Z63) from the NCBI Molecular Modeling Database (MMDB), which is accessible.^10^ Next, we performed ligand-receptor docking analysis using the ClusPro 2.0 online server, which you can find (41).^11^ We then examined the interaction surfaces of the resulting complexes using PDBE Pisa, available (42).^12^ Finally, we visualized the complexes using PyMOL software and analyzed them using Ligplot+ software.

### 2.10. Molecular dynamics simulation

We performed molecular dynamics simulations of the MEV-Fc-TLR2 and MEV-Fc-TLR4 complexes using the iMODSweb server. This server, which is accessible,^13^ allows us to explore possible trajectories between two conformations and enables interactive analysis of the resulting structures, animations, and trajectories in three dimensions. Importantly, it can effectively simulate and explore even large molecules (43).

### 2.11. Immune simulation

We used the C-ImmSim server, which you can access,^14^ to predict the ability of MEV-Fc to stimulate the production of specific antibodies and various cytokines by immune cells (44). This server is capable of predicting the immune response of both B lymphocytes and T lymphocytes, including Th1 and Th2 lymphocytes.

In our simulation, we considered the recommended minimum interval between the initial and subsequent doses of most vaccines, which is 28 days (45). Therefore, we configured the simulation parameters as follows: three injections, with each injection spaced 28 days apart; a random seed value set to 12,345; a simulation volume of 50; and a total of 1,050 simulation steps. We kept the remaining parameters at their default values.

### 2.12. *In silico* cloning

To obtain an optimized nucleotide sequence, we used the Java Codon Adaptation Tool (JCat), which is available.^15^ This tool quickly generates optimized codon sequences tailored to the chosen expression host, thereby enhancing the production yield of heterologous proteins (46). In this case, we selected *E. coli* (strain K12) as the preferred bacterial strain. The ideal Codon Adaptation Index (CAI) value is 1, and the GC content percentage should ideally fall within the range of 30 to 70%.

After obtaining the optimized gene sequence, we cloned it into the expression vector pET28a(+) using Hind III and BamHI digestion sites. Subsequently, we analyzed the cloned sequence using Snapgene software.

## 3. Results

### 3.1. Selection and construction of immunodominant epitopes

**Figure 2:**
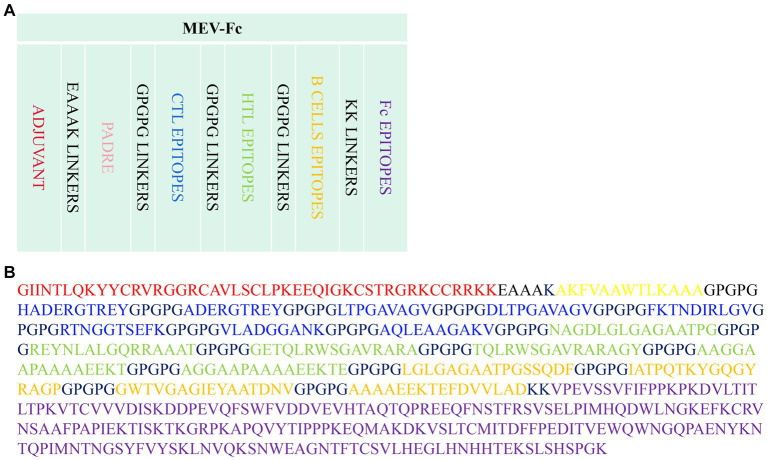
**(A)** Schematic diagram of the MEV-Fc construct. **(B)** Amino acid sequence of the MEV-Fc construct.

A set of four proteins was retrieved from the NCBI server, and subsequently, their antigenicity was predicted using VaxiJen (Table 1). Utilizing the IEDB MHCI and MHC II servers, we predicted a total of 308 CTL epitopes (Supplementary Table 1) and 89 HTL epitopes (Supplementary Table 2) with a percentile rank of 0.5. Among the 308 CTL epitopes, 57 epitopes with immunogenicity >0 and an antigenicity score > 1 were selected (Supplementary Table 3). Additionally, from the 89 HTL epitopes, 28 epitopes exhibiting positive induction of IFN-γ and an antigenicity score > 0 were selected (Supplementary Table 4). The ABCpred server was employed to predict 68 linear B cell epitopes (Supplementary Table 5). Ultimately, 18 epitopes were chosen as candidates for vaccine construction (Table 2), including eight epitopes with the highest immunogenicity or highest antigenicity score, six HTL epitopes with the highest antigenicity score or highest IFN-γ scores, and four B cell epitopes with the highest ABCpred prediction scores. The predicted and screened CTL epitopes, HTL epitopes, and linear B-cell epitopes were linked together using GPGPG linkers. The hBD-3 sequences and PADRE sequences were connected via EAAAK linkers, while the fc (PDB ID: 1KJ6) sequences were linked using KK linkers. The resulting peptide molecule was named MEV-Fc, and its schematic diagram and amino acid sequence are presented in Figure 2.

**Table 2:** Final selection of HEL, CTL, B-cell peptides.

Protein name	CTL epitope	HTL epitope	B-cell epitope
L7/L12	VLADGGANK	AAGGAAPAAAAEEKT	AAAAEEKTEFDVVLAD
	AQLEAAGAKV	AGGAAPAAAAEEKTE	–
OMP16	HADERGTREY	NAGDLGLGAGAATPG	LGLGAGAATPGSSQDF
	ADERGTREY	REYNLALGQRRAAAT	–
OMP19	LTPGAVAGV	–	IATPQTKYGQGYRAGP
	DLTPGAVAGV	–	–
OMP25	FKTNDIRLGV	GETQLRWSGAVRARA	GWTVGAGIEYAATDNV
	RTNGGTSEFK	TQLRWSGAVRARAGY	–

### 3.2. Prediction of physicochemical properties, antigenicity, immunogenicity, sensitization, and toxicity of MEV-fc

MEV-Fc, a protein we studied, has some important characteristics. It is composed of 600 amino acids and has a molecular weight of about 61.2 kilodaltons. Its isoelectric point (PI) is approximately 8.89, indicating its charge at a specific pH. It is relatively stable with an instability index of 24.06 and Aliphatic index of 57.35. In terms of its lifespan, MEV-Fc is estimated to last about 30 h in mammalian reticulocytes *in vitro*. It has a slight preference for water, making it slightly hydrophilic with a GRAVY value of −0.500 (Table 3). Moreover, it is highly likely to be soluble during production, with a probability of 0.976819.

**Table 3:** Physicochemical properties predicted by Expasy Protparam server.

Number of amino acids	Molecular weight	Theoretical pI	Estimated half-life	Instability index	Aliphatic index	GRAVY
600	61202.56 Da	8.89	30 h (mammalian reticulocytes, *in vitro*). >20 h (yeast, *in vivo*); >10 h (*Escherichia coli*, *in vivo*)。	24.06	57.35	−0.5

Regarding its potential as an antigen, predictions suggest it has a high likelihood of triggering an immune response with an immunogenicity score of 4.18 and good antigenicity (1.0342). Importantly, MEV-Fc is considered non-sensitizing and non-toxic based on prediction, indicating its safety profile.

In summary, MEV-Fc possesses favorable characteristics, including stability, solubility, and the potential to elicit an immune response, making it a strong candidate for a peptide vaccine. Additionally, it is considered safe for use.

### 3.3. Prediction and validation of secondary and tertiary structures of MEV-fc

**Figure 3:**
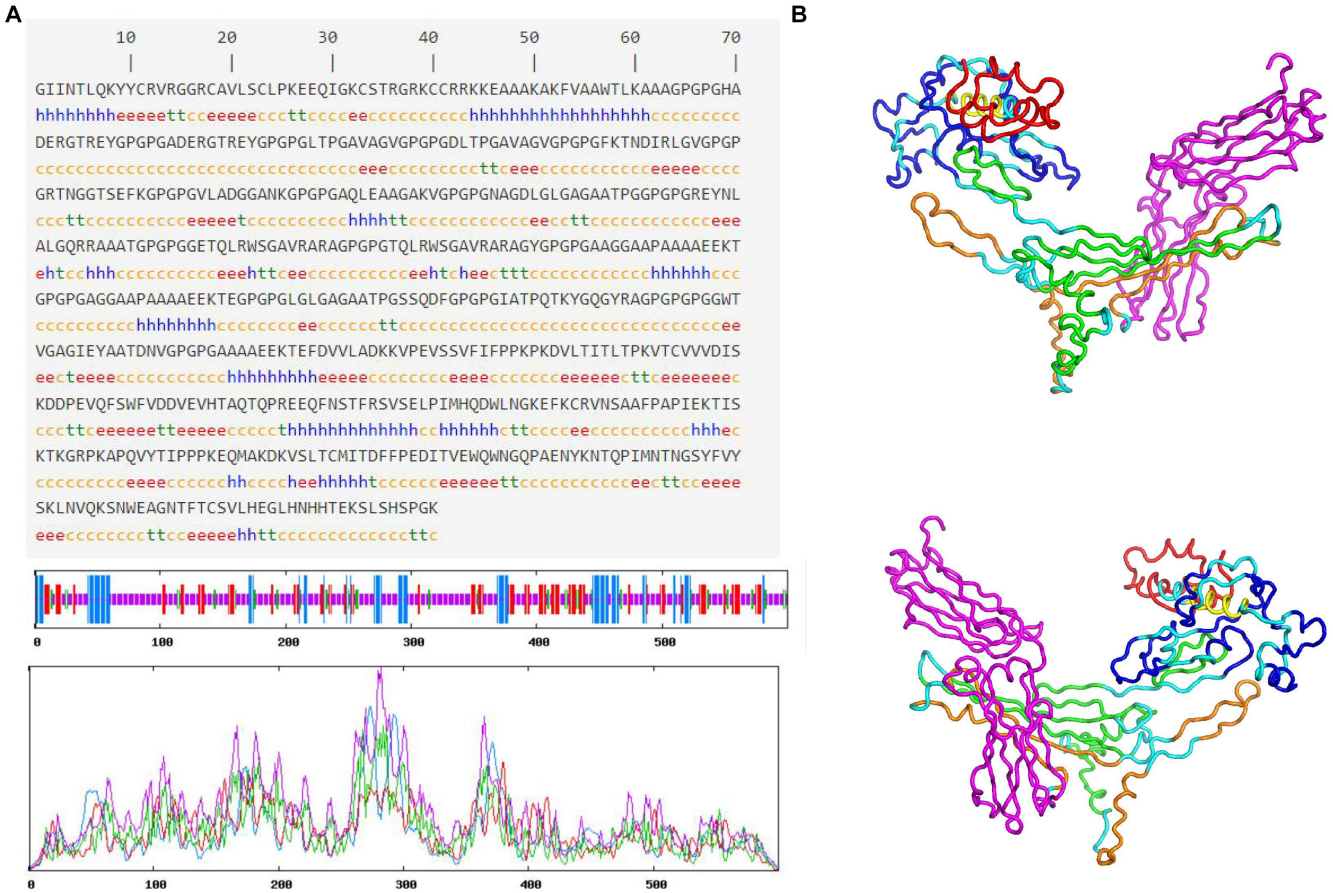
Prediction of MEV-Fc secondary and tertiary structures. **(A)** The prediction of MEV-Fc secondary structure. **(B)** The 3D model showcases the MEV-Fc tertiary structure, providing both front view and back view perspectives.

**Figure 4:**
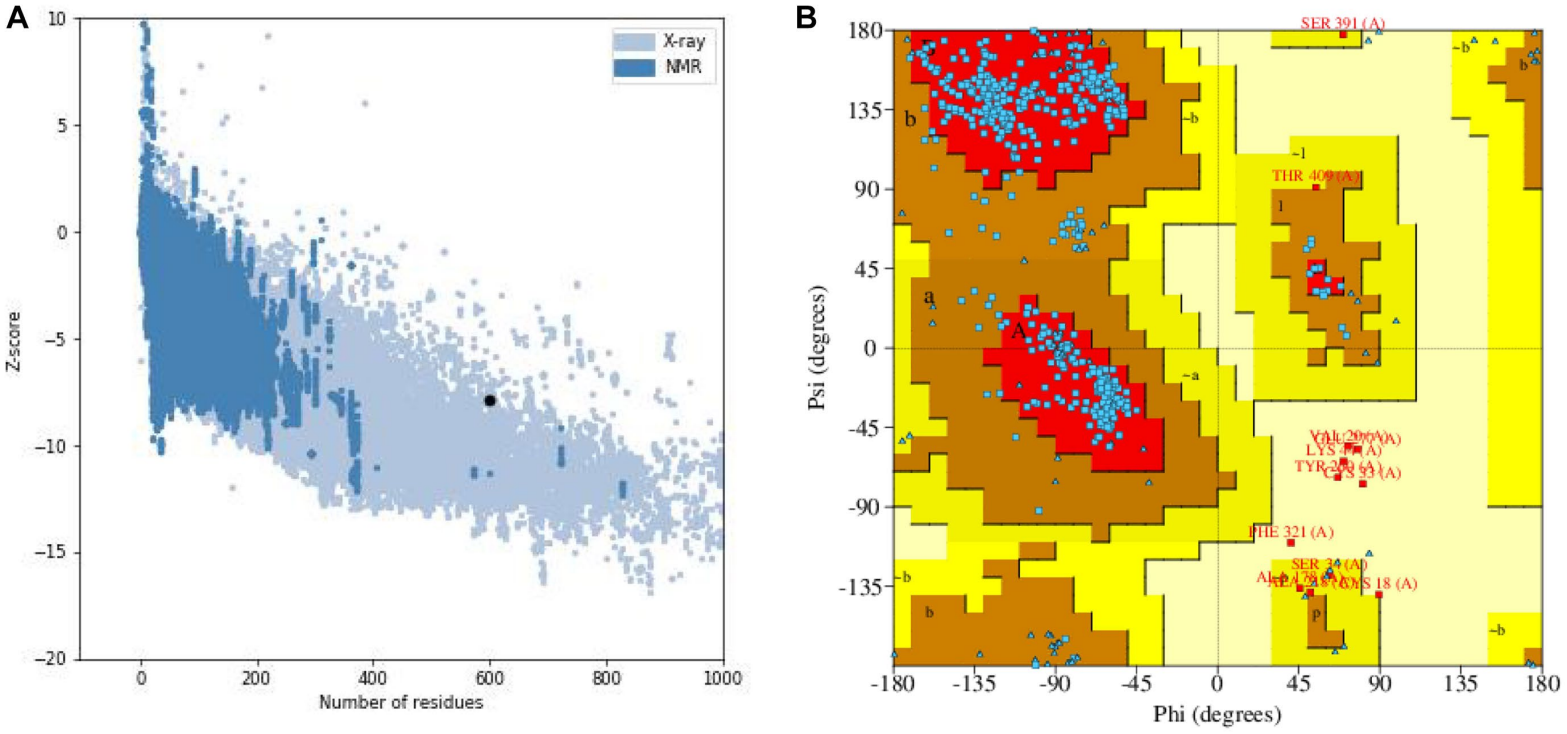
**(A)** Z-score evaluation results: the x-axis represents the number of amino acids in the protein, while the y-axis represents the score. The figure displays regions marked by blue and gray spots, which indicate the expected scoring range for proteins. The black spots represent the target proteins. **(B)** Ramachandran plot: the plot illustrates the distribution of residues based on their conformational angles.

The results of the secondary structure prediction (Figure 3A) show that MEV-Fc’s conformation consists of 15.33% alpha helix (Hh), 19.17% extended strand (Ee), 7.17% beta-turn (Tt), and 58.33% random coil (Cc). To predict the tertiary structure of MEV-Fc, we used the Robetta server, and the resulting model was visualized using the Pymol software package (Figure 3B). To assess the model’s quality, we conducted ProSA-web analysis, which yielded a Z-Score of −7.88 (Figure 4A). This Z-Score falls within the normal range for native proteins of similar size, confirming the credibility of the model. Additionally, we generated a Ramachandran plot using the PROCHECK server to comprehensively evaluate the overall quality of the structural arrangement (Figure 4B). The analysis showed that 87.1% of residues were in the most favored regions, 10.2% in additional allowed regions, 1.2% in generously allowed regions, and 1.6% in disallowed regions.

In conclusion, the ERRAT server indicated an overall quality factor of 86.531, surpassing the generally accepted threshold of 50 for a good model. Therefore, we can confidently consider the tertiary structure of MEV-Fc as a reliable and accurate model.

### 3.4. Prediction of B-cell epitopes

Using the ElliPro server, we identified a comprehensive set of 311 residues that make up discontinuous B cell epitopes (Supplementary Table 6). These epitopes had scores ranging from 0.524 to 0.854. Additionally, our prediction revealed 18 consecutive B cell epitopes (Supplementary Table 7), which varied in size from 4 to 42 residues, with scores ranging from 0.512 to 0.832.

### 3.5. Molecular docking

**Figure 5:**
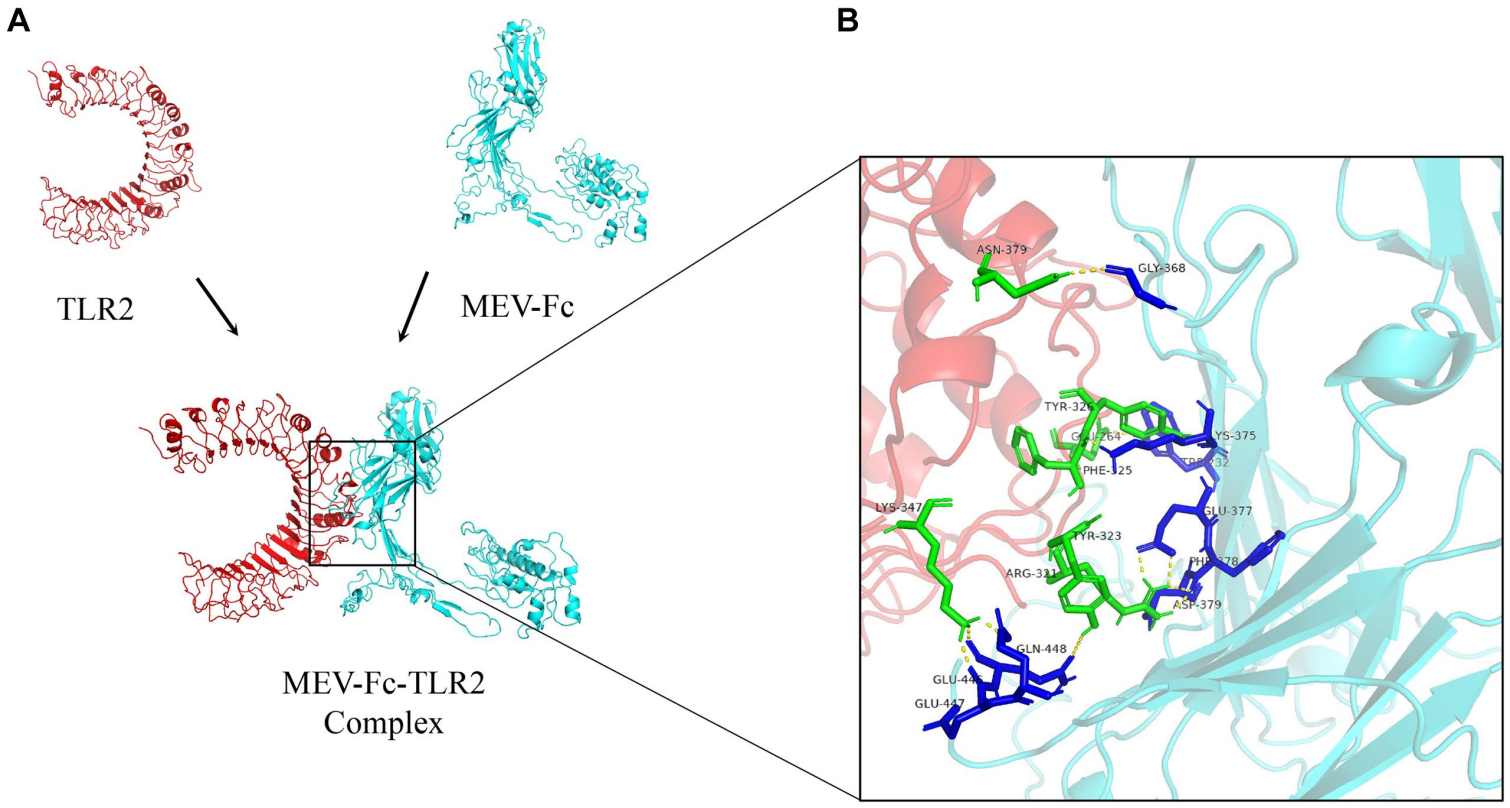
**(A)** Outcome plots derived from the ClusPro molecular docking of the vaccine structures (cyan) and TLR2 receptors (red). **(B)** Analysis of interactions within the MEV-Fc-TLR2 complex and generation of their 3D images utilizing the PyMol visualization tool.

**Figure 6:**
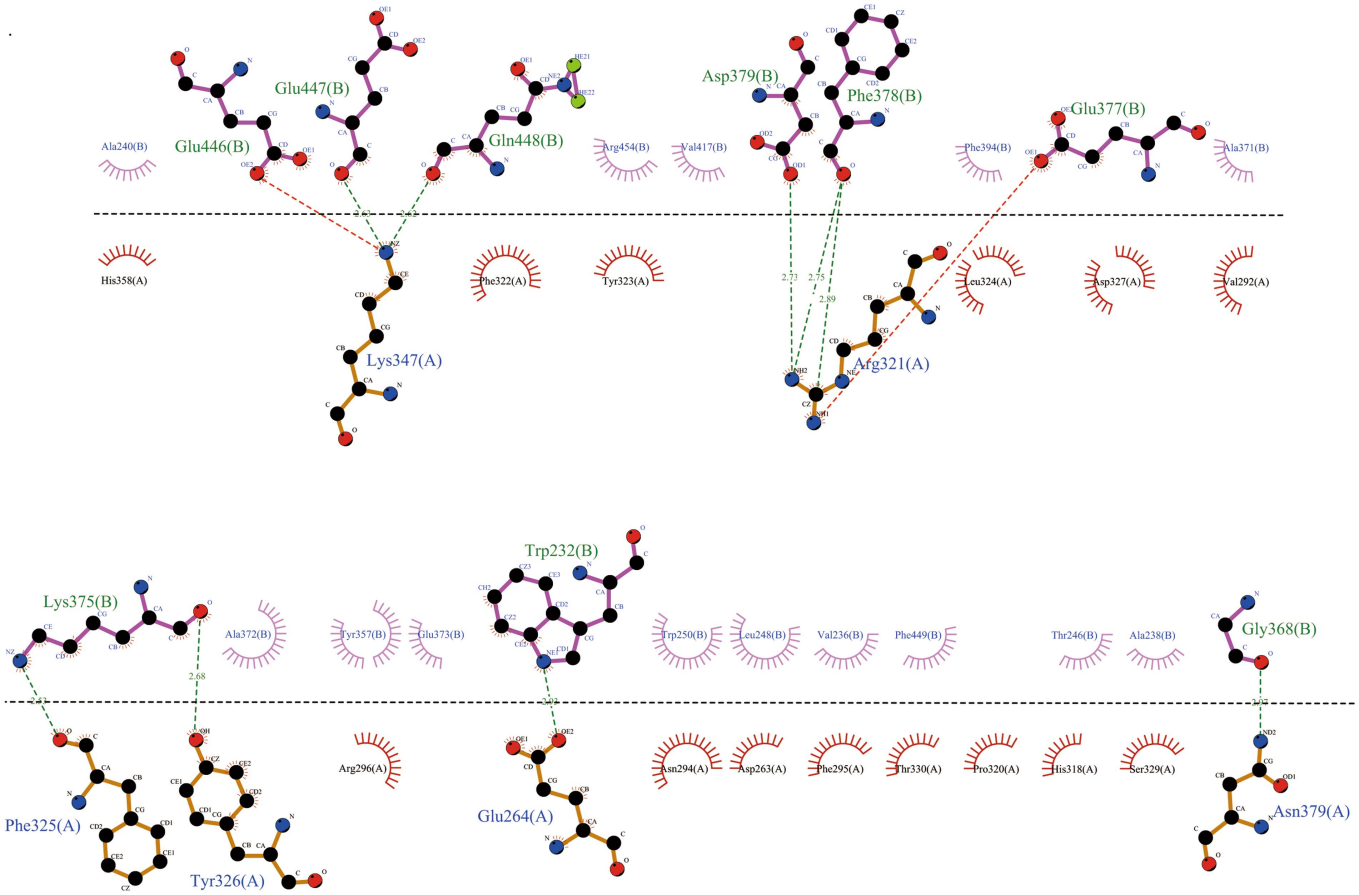
Further analysis of the interactions within the MEV-Fc-TLR2 complex and generation of their two-dimensional images using the Ligplot+ visualization tool.

We used a powerful tool called ClusPro 2.0 to understand how our vaccine interacts with TLR2 and TLR4, two important proteins. When we examined how the vaccine connects with TLR2, we got 10 different models. We found that one model stood out, showing 11 hydrogen bonds and 5 salt-bridge interactions (Figure 5; Supplementary Table 8). The strength of the bond, called binding free energy, was −10.4 kcal/mol, indicating a strong connection between MEV-Fc and TLR2. To better understand this interaction, we used Ligplot+ to create a clear 2D picture of how they bond (Figure 6).

**Figure 7:**
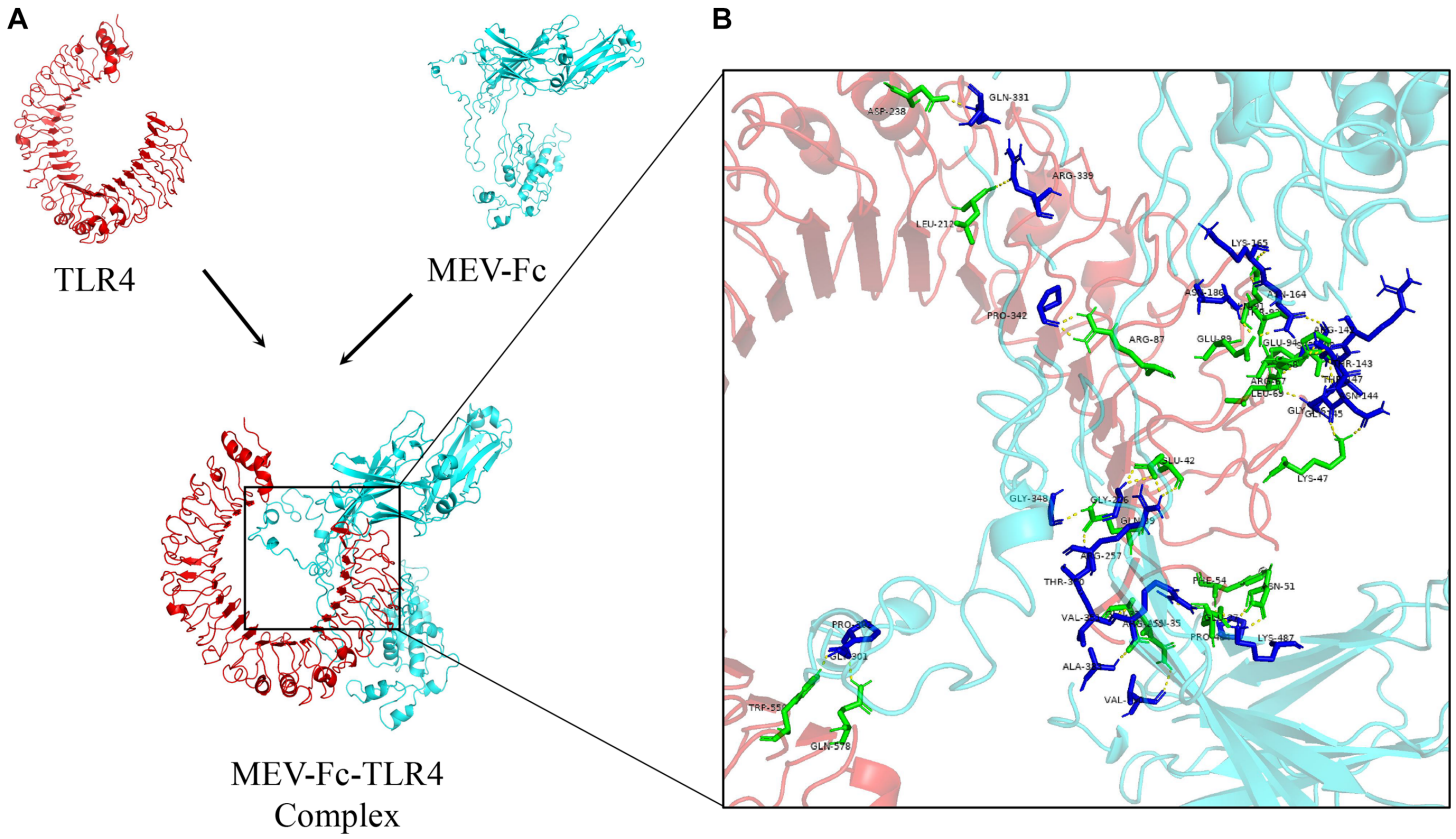
**(A)** Result plots generated from the molecular docking of Cluspro, displaying the vaccine structures in cyan and TLR4 receptors in red. **(B)** Analysis of the MEV-Fc-TLR4 complex interactions and generation of their 3D images using the PyMol visualization tool.

**Figure 8:**
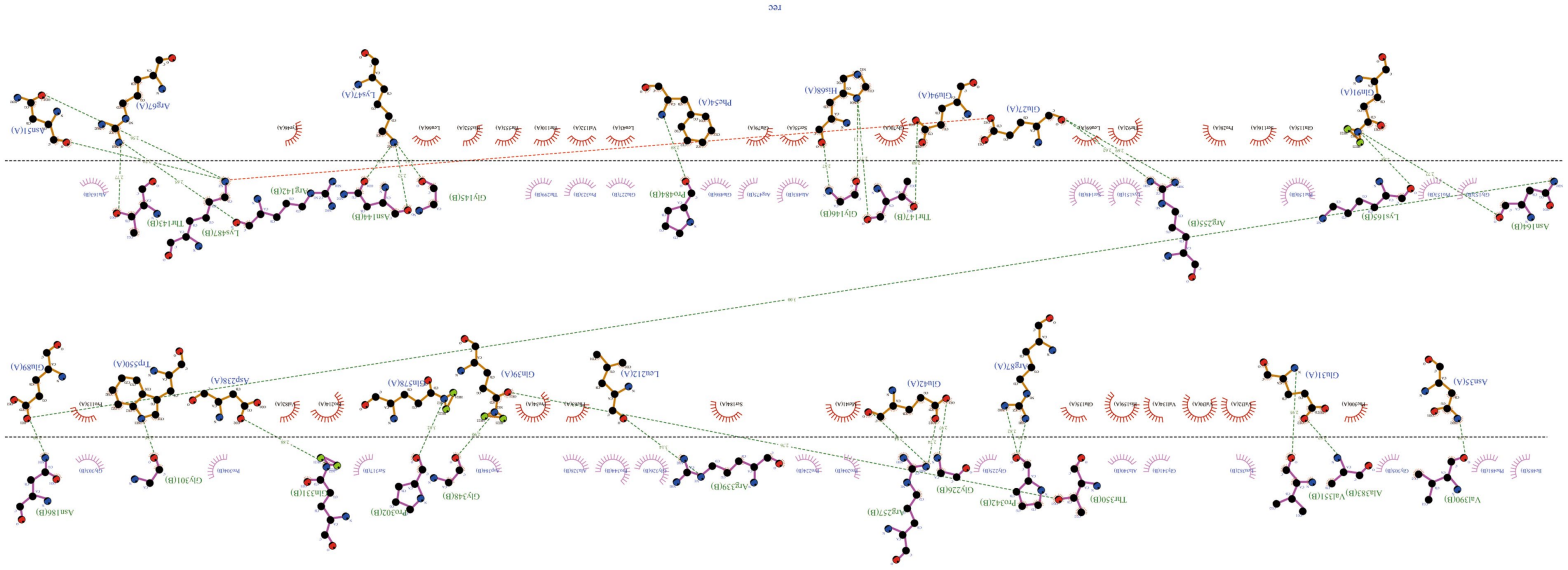
Analysis of the MEV-Fc-TLR4 complex interactions using the Ligplot+ visualization tool, resulting in two-dimensional images of the interactions.

In a similar way, we looked at how our vaccine interacts with TLR4. We also got 10 different models. One model showed an impressive 27 hydrogen bonds and 5 salt-bridge interactions (Figure 7; Supplementary Table 9). The strength of this bond, in terms of binding free energy, was even higher at −18.8 kcal/mol, suggesting a very strong link between MEV-Fc and TLR4. We used Ligplot+ again to create a clear 2D picture of this complex bond (Figure 8).

### 3.6. Molecular dynamics simulation between MEV-fc and TLR

**Figure 9:**
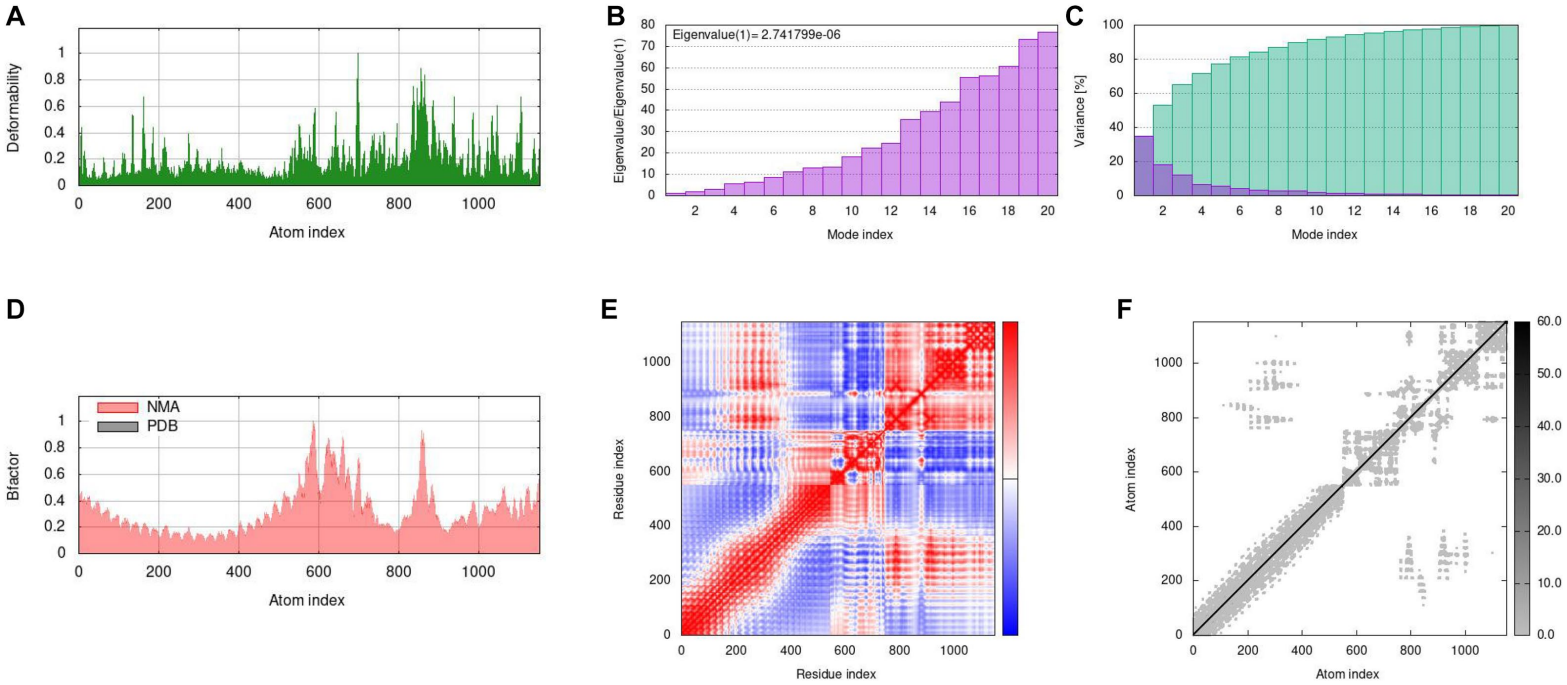
Molecular dynamics simulation results of MEV-Fc-TLR2. **(A)** Deformability analysis. **(B)** Eigenvalues of the simulation. **(C)** Variance plots, indicating individual (red) and cumulative (green) variances. **(D)** B-factor comparison. **(E)** Covariance map showing correlated (red), uncorrelated (white), and anticorrelated (blue)movements. **(F)** Elastic network representation, with darker gray areas indicating higher rigidity.

We used a tool called IModS to simulate how the molecules in our vaccine move and behave. In Figures 9 A-F, we show the results of this simulation. In Figure A, we can see how our vaccine (MEV-Fc) behaves when it is connected to TLR2. We used a method called normal mode analysis (NMA) to understand how flexible the proteins are. Figure B shows specific values related to this analysis for the MEV-Fc-TLR2 complex. In Figure C, variance plots showed a cumulative or individual variance of MEV-Fc-TLR2 complex with green or purple, respectively. In Figure D, we compare the actual movement (B factor) with what we predicted through NMA. Figure E displays a map that highlights how different parts of the complex move together. The red areas show significant motions happening together. Lastly, Figure F provides a diagram showing how the parts of the docked protein complexes are interconnected, like springs with varying stiffness. Darker areas indicate stiffer connections. Similar results were obtained for TLR4 (Supplementary Figure 1).

### 3.7. Immune simulation

**Figure 10:**
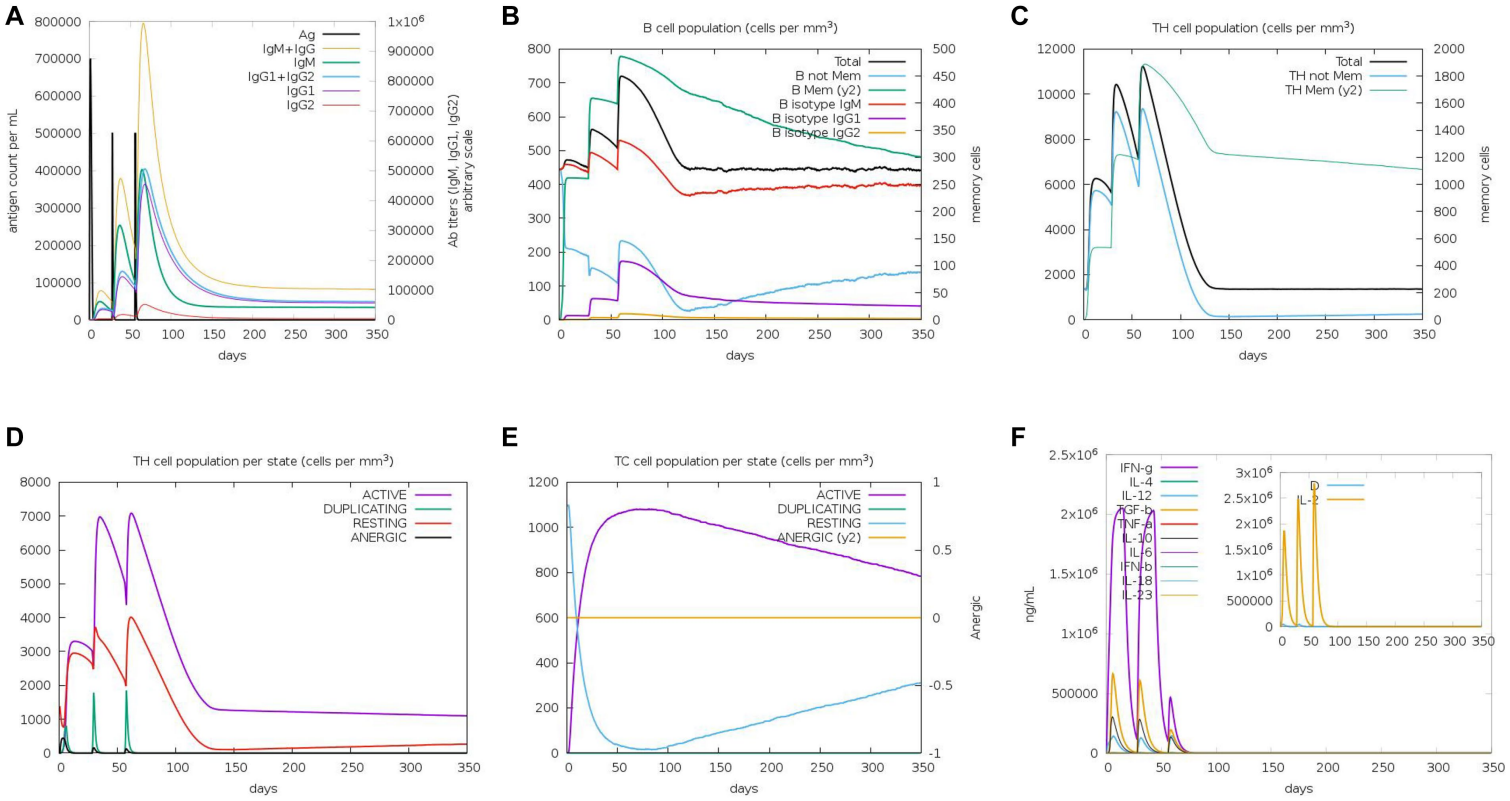
Immune simulation. **(A)** Levels of immunoglobulins in different states after antigen stimulation. **(B)** Distribution of B-cell subtypes in different states after antigen stimulation. **(C)** Count of CD4 T-helper lymphocytes after antigen stimulation. **(D)** Distribution of CD4 T-helper lymphocytes divided by activation status after antigen stimulation. **(E)** Count of CD8 T-cytotoxic lymphocytes divided by activation status. **(F)** Production of multiple cytokines following antigen stimulation.

We used a tool called C-ImmSim to predict how the immune system in mice would respond to the MEV-Fc vaccine. Here are the key findings (Figure 10): (1) Antibody Levels: The results showed that the levels of IgM and IgG antibodies increased progressively after three rounds of vaccine injections (Figure 10A). This suggests that the vaccine triggered an antibody response in mice. (2) B-Cells: B-cells, which are responsible for producing antibodies, became more active with each vaccine injection, reaching their highest level after the final injection (Figure 10B). (3) Helper T Cells (TH-Cells): Both the total and memory populations of helper T cells (TH-cells) increased significantly (Figure 10C). The active TH-cell population also expanded robustly, reaching its peak after the third immunization (Figure 10D). (4) Cytotoxic T Cells (TC Cells): The count of active cytotoxic T lymphocytes (TC cells) increased gradually after each immunization injection (Figure 10E). (5) Cytokines: There was a notable increase in the levels of interferon-γ and IL-2, which are immune system signaling molecules, in response to antigen stimulation (Figure 10F). These findings suggest that the MEV-Fc vaccine could elicit a strong and diverse immune response in mice, involving antibodies, B-cells, helper T cells, cytotoxic T cells, and cytokines.

### 3.8. *In silico* cloning

**Figure 11:**
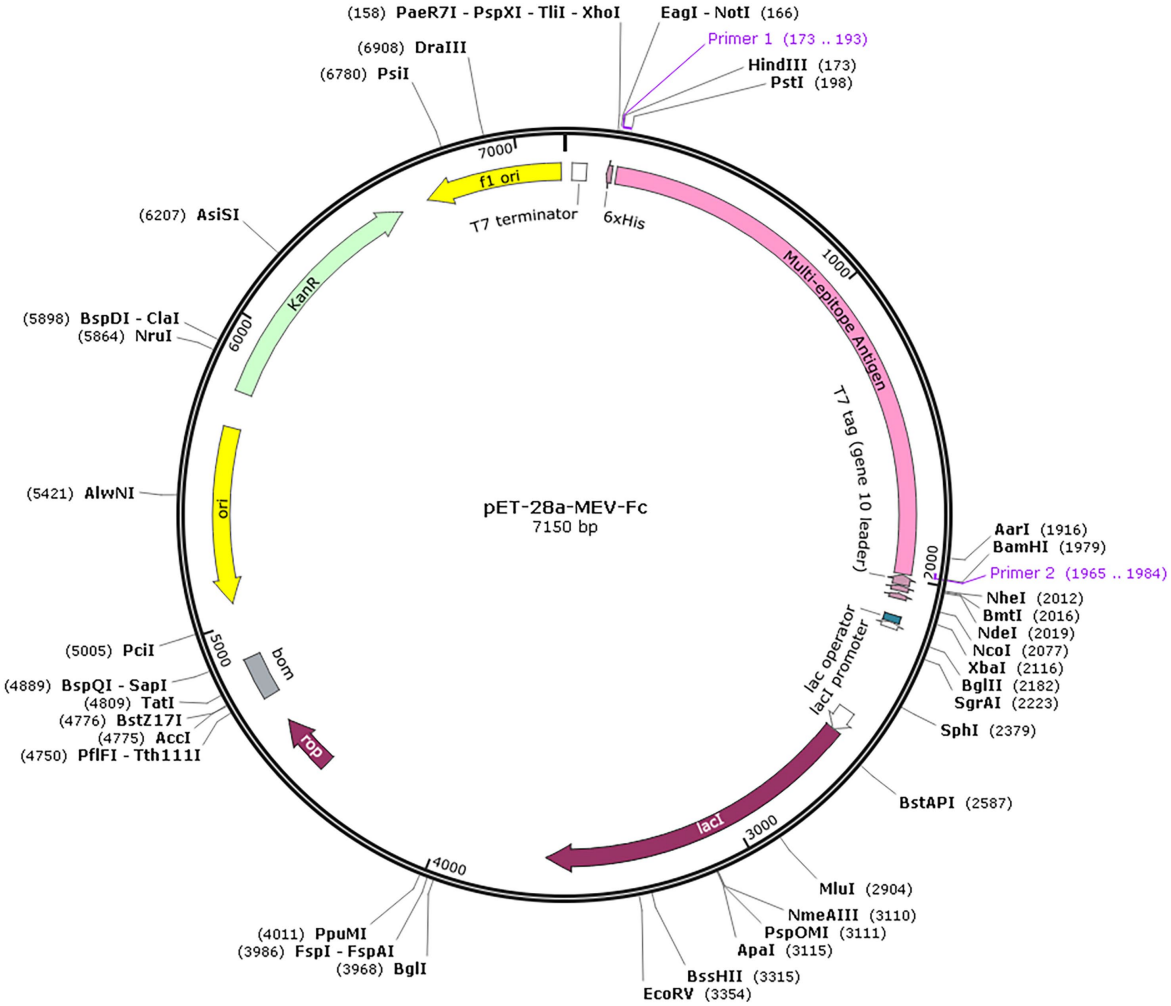
*In silico* cloning. The multi-epitope vaccine sequence (highlighted in pink) was successfully inserted into the pET28a (+) expression vector at the HindIII and BamHI restriction endonuclease cleavage sites.

Codon optimization is a technique used to improve the way a particular genetic sequence is expressed by enhancing translational efficiency (47). In this context, we optimized the codon usage in the MEV-Fc construct sequence using a tool called JCat. This optimization resulted in a Codon Adaptation Index (CAI) value of 1, and the GC content was measured at 56.99%, which falls within the desirable range. These findings suggest that the optimized sequence is likely to be efficiently expressed in the *E. coli* expression system. You can see the result in (Figure 11), which shows the expression vector pET 28a(+) with the inserted fragment of the multi-epitope vaccines.

## 4. Discussion

Brucellosis is a complex disease that poses a significant health risk to both humans and livestock (48). Vaccines are considered the most cost-effective way to prevent diseases caused by infectious agents (49). Subunit vaccines, in particular, show promise due to their safety, non-infectious nature, inability to revert to a virulent form, and precise control over desired effects (50). However, developing a safe and effective subunit vaccine against *Brucella* for use in humans and animals has been challenging (51, 52). Advances in bioinformatics, structural biology, and computational tools have transformed vaccine design (53). Bioinformatics has been used extensively to predict and design vaccines for various pathogens, including bacteria, viruses, fungi, and cancer (54).

In this context, we focused on *Brucella abortus* proteins OMP16, OMP19, OMP25, and L7/L12, which are known for their strong immunogenicity and potential to induce immune protection. We developed a multi-epitope vaccine based on these proteins and confirmed its immunogenicity. This vaccine has the potential to provide broad protection against *Brucella* infection.

*Brucella*, an intracellular and facultative intracellular parasite, has the ability to replicate within specialized or nonprofessional phagocytes (55). It has been demonstrated that cell-mediated immunity, particularly involving macrophages and T cells, plays a crucial role in immunoprotection against *Brucella* and other intracellular bacterial pathogens (56). B lymphocytes, are pivotal components of humoral immunity, producing antigen-specific antibodies that play a critical role in eliminating *Brucella* infection (57, 58). Therefore, an ideal anti-*Brucella* vaccine should encompass both T-cell and B-cell epitopes. In our study, we employed epitope prediction techniques to identify CD4+ and CD8+ short peptide sequences within the antigen, targeting both CTL and HTL epitopes (59). B cell epitopes can be recognized and bind to antibodies (on the surface of B cells or free antibodies), stimulating the immune system’s response against pathogens (38). By incorporating B cell epitopes, the vaccine can more effectively activate B cells and induce humoral immunity. Consequently, utilizing the IEDB server, we identified 8 dominant CTL epitopes and 6 dominant HTL epitopes from the 4 proteins. Additionally, the ABCpred server was employed to identify 4 dominant linear B cell epitopes present in all four proteins.

The choice of a suitable linker is crucial when combining different components in a vaccine (60). It helps prevent unintended interactions and improves how the vaccine is processed and presented to the immune system. In our vaccine design, we added the Fc sequence to the vaccine to address the issue of the vaccine’s short lifespan and prevent its breakdown in the body. Fc fragment fusion proteins have been studied as potential vaccines or treatments for various diseases like influenza, tuberculosis, and swine fever (61–63). While Fc fragments can help with antigen presentation by binding to receptors on immune cells, this may not be enough to activate certain immune cells and promote the desired immune response. To boost the immune response, we paired our vaccine with adjuvants and molecular agonists (64). We chose β-defensin as an adjuvant because it has antimicrobial properties and can modulate the immune system (65). Additionally, we included the PADRE sequence, which acts as an activator of certain immune receptors, to enhance the vaccine’s long-term effectiveness (66). With these modifications, we successfully created a peptide vaccine consisting of 600 amino acids.

We conducted further analysis to assess the physical and chemical properties, sensitization, and toxicity of MEV-Fc. Previous research suggests that vaccine proteins should have a molecular mass of less than 110 kDa (67). MEV-Fc has a predicted molecular weight of about 61.2 kDa, which falls within this desirable range. It also shows good antigenic properties, stability, hydrophilicity, solubility, and low sensitivity with an antigenicity score of 1.0342.

When we looked at its secondary structure, we found that MEV-Fc is composed of 15.33% alpha helix, 7.17% beta turns, and 58.33% random coils. These structural elements play a crucial role in how antibodies recognize the vaccine when the body is infected (40). The presence of beta turns and random coils in protein vaccines helps create antigenic epitopes (68).

We also generated a 3D model of the *Brucella* vaccine using the Robetta server. The Z score of −7.88 indicates that the protein structure prediction is accurate. In a Ramachandran plot analysis, over 98% of residues were located in favorable and permissive regions. This confirms the high accuracy and confidence of the predicted tertiary structure of MEV-Fc, making it a strong foundation for an effective vaccine model.

Protein–protein docking has become an important tool in the fields of immunoinformatics and pharmacological research. TLR2 and TLR4 are particularly important because they play a crucial role in generating specific T cells and are essential for the body’s defense against *Brucella* infection (69, 70). Therefore, we selected TLR2 and TLR4 as the receptors for molecular docking with MEV-Fc. Through molecular docking and subsequent molecular dynamics simulations, the stability of the MEV-Fc-TLR complex interactions was assessed. The docking results revealed the presence of an atomistic interaction interface between MEV-Fc and TLR2/TLR4, indicating a strong interaction between them, it indicates that MEV-Fc can activate the TLR2/TLR4 receptor signaling pathway and activate the immune response.

As mentioned earlier, *Brucella* is a type of bacteria that can survive and multiply within cells. To combat this, stimulating a T-cell-dependent immune response is crucial, as it hinders the bacteria’s growth within cells (71). B cells are also vital for the body’s defense by producing specific proteins that aid in immunity (59). The predictions regarding MEV-Fc showed that it effectively triggered both the innate and adaptive immune responses in mice. Notably, it increased various cell populations related to immunity and promoted the production of antibodies (Ig). The predicted results also indicated a significant rise in interferon-gamma and IL-2 levels. Interferon-gamma is important for fighting bacterial infections as it activates macrophages, aiding in the elimination of intracellular pathogens (72).

To ensure effective transcription and translation, we used JCAT software for codon optimization. The goal was to predict the best way to express the MEV-Fc construct in the *E. coli* K12 strain. The results showed a CAI value of 1 and a GC content of 56.99%. This suggests a high chance of successful and efficient expression in the *E. coli* expression system. Experimental verification of the immunogenicity and safety of the vaccine candidates designed and constructed in this study is required. The next step in this study will therefore be to perform high throughput cloning of the constructed vaccine candidates, expression of the purified recombinant proteins, immunization of the animals, and immunological assessments to ensure the true potential of the designed *Brucella* epitope vaccine.

## 5. Conclusion

In summary, this study introduces a novel vaccine candidate called MEV-Fc, designed specifically to prevent brucellosis. MEV-Fc includes 8 CTL epitopes, 6 HTL epitopes, 4 B cell epitopes, along with adjuvants and Fc fragments. MEV-Fc shows strong antigenicity and immunogenicity, and it is safe with no sensitizing or toxic effects. Importantly, MEV-Fc has a strong affinity for both TLR2 and TLR4, indicating a stable interaction. Additionally, MEV-Fc triggers a robust innate and adaptive immune response, leading to increased levels of Th1-type cytokines like interferon-gamma and IL-2. These promising findings suggest that MEV-Fc could be an excellent vaccine candidate for preventing brucellosis. But *in vivo* studies and laboratory data are also required to confirm vaccine efficacy.

## Data availability statement

The datasets presented in this study can be found in online repositories. The names of the repository or repositories and accession number(s) can be found in the Supplementary material.

## Author contributions

JS and JY presented the concept and edited the article. AW and YW presented the concept, analyzed the data, and wrote the manuscript. AA and ZX designed the figures and edited the Manuscript. DZ and KZ review the manuscript and contributed to the data analysis. All authors contributed to the article and approved the submitted version.

## Footnotes

^1^http://tools.iedb.org/mhci/

^2^http://tools.iedb.org/mhcii/

^3^https://webs.iiitd.edu.in/raghava/abcpred/ABC_submission.html

^4^https://www.expasy.org/resources/protparam

^5^http://scratch.proteomics.ics.uci.edu//

^6^http://ddg-pharmfac.net/AllergenFP/

^7^http://crdd.osdd.net/raghava/toxinpred/

^8^https://saves.mbi.ucla.edu/

^9^http://services.mbi.ucla.edu/ERRAT/

^10^https://www.ncbi.nlm.nih.gov/structure/

^11^https://cluspro.bu.edu/login.php?redir=/home.php

^12^https://www.ebi.ac.uk/pdbe/pisa/

^13^https://imods.iqfr.csic.es/

^14^https://150.146.2.1/C-IMMSIM/index.php

^15^http://www.jcat.de/

## References

[1] Byndloss MX Tsolis RM. Brucella spp. virulence factors and immunity. Ann Rev Anim Biosci. (2016) 4:111–27. doi: 10.1146/annurev-animal-021815-111326 26734887

[2] Boschiroli ML Foulongne V O'Callaghan D. Brucellosis: a worldwide zoonosis. Curr Opin Microbiol. (2001) 4:58–64. doi: 10.1016/s1369-5274(00)00165-x 11173035

[3] Celli J. The changing nature of the Brucella-containing vacuole. Cell Microbiol. (2015) 17:951–8. doi: 10.1111/cmi.12452, PMID: 25916795 PMC4478208

[4] Lalsiamthara J Lee JH. Brucella lipopolysaccharide reinforced Salmonella delivering Brucella immunogens protects mice against virulent challenge. Vet Microbiol. (2017) 205:84–91. doi: 10.1016/j.vetmic.2017.05.012, PMID: 28622869

[5] Godfroid J Scholz HC Barbier T Nicolas C Wattiau P Fretin D. Brucellosis at the animal/ecosystem/human interface at the beginning of the 21st century. Prev Vet Med. (2011) 102:118–31. doi: 10.1016/j.prevetmed.2011.04.007 21571380

[6] Dorneles EMS Sriranganathan N Lage AP. Recent advances in *Brucella abortus* vaccines. Vet Res. (2015) 46:76. doi: 10.1186/s13567-015-0199-7, PMID: 26155935 PMC4495609

[7] Avila-Calderón ED Lopez-Merino A Sriranganathan N Boyle SM Contreras-Rodríguez A. A history of the development of Brucella vaccines. Biomed Res Int. (2013) 2013:743509. doi: 10.1155/2013/743509, PMID: 23862154 PMC3686056

[8] Tibor A Decelle B Letesson JJ. Outer membrane proteins Omp10, Omp16, and Omp19 of Brucella spp. are lipoproteins. Infect Immun. (1999) 67:4960–2. doi: 10.1128/iai.67.9.4960-4962.1999, PMID: 10456959 PMC96837

[9] Godlewska R Wiśniewska K Pietras Z Jagusztyn-Krynicka EK. Peptidoglycan-associated lipoprotein (pal) of gram-negative bacteria: function, structure, role in pathogenesis and potential application in immunoprophylaxis. FEMS Microbiol Lett. (2009) 298:1–11. doi: 10.1111/j.1574-6968.2009.01659.x, PMID: 19519769

[10] Alizadeh H Dezfulian M Rahnema M Fallah J Esmaeili D. Protection of BALB/c mice against pathogenic Brucella abortus and *Brucella melitensis* by vaccination with recombinant Omp16. Iran J Basic Med Sci. (2019) 22:1302–7. doi: 10.22038/ijbms.2019.36369.8665 32128095 PMC7038423

[11] Pasquevich KA Carabajal MV Guaimas FF Bruno L Roset MS Coria LM. Omp19 enables *Brucella abortus* to evade the antimicrobial activity from Host's proteolytic Defense system. Front Immunol. (2019) 10:1436. doi: 10.3389/fimmu.2019.01436, PMID: 31297115 PMC6607954

[12] Barrionuevo P Cassataro J Delpino MV Zwerdling A Pasquevich KA García Samartino C. *Brucella abortus* inhibits major histocompatibility complex class II expression and antigen processing through interleukin-6 secretion via toll-like receptor 2. Infect Immun. (2007) 76:250–62. doi: 10.1128/iai.00949-07, PMID: 17984211 PMC2223644

[13] Pasquevich KA Ibañez AE Coria LM García Samartino C Estein SM Zwerdling A. An oral vaccine based on U-Omp19 induces protection against *B. abortus* mucosal challenge by inducing an adaptive IL-17 immune response in mice. PLoS One. (2011) 6:e16203. doi: 10.1371/journal.pone.0016203 21264260 PMC3021544

[14] Cloeckaert A Vizcaíno N Paquet J-Y Bowden RA Elzer PH. Major outer membrane proteins of Brucella spp.: past, present and future. Vet Microbiol. (2002) 90:229–47. doi: 10.1016/s0378-1135(02)00211-0, PMID: 12414146

[15] Jubier-Maurin V Boigegrain RA Cloeckaert A Gross A Alvarez-Martinez MT Terraza A. Major outer membrane protein Omp25 of *Brucella suis* is involved in inhibition of tumor necrosis factor alpha production during infection of human macrophages. Infect Immun. (2001) 69:4823–30. doi: 10.1128/iai.69.8.4823-4830.2001, PMID: 11447156 PMC98570

[16] Commander NJ Spencer SA Wren BW MacMillan AP. The identification of two protective DNA vaccines from a panel of five plasmid constructs encoding *Brucella melitensis* 16M genes. Vaccine. (2006) 25:43–54. doi: 10.1016/j.vaccine.2006.07.046, PMID: 17049676

[17] Goel D Bhatnagar R. Intradermal immunization with outer membrane protein 25 protects Balb/c mice from virulent *B. abortus* 544. Mol Immunol. (2012) 51:159–68. doi: 10.1016/j.molimm.2012.02.126 22464098

[18] Golshani M Rafati S Siadat SD Nejati-Moheimani M Shahcheraghi F Arsang A. Improved immunogenicity and protective efficacy of a divalent DNA vaccine encoding Brucella L7/L12-truncated Omp31 fusion protein by a DNA priming and protein boosting regimen. Mol Immunol. (2015) 66:384–91. doi: 10.1016/j.molimm.2015.04.015, PMID: 25968974

[19] Oliveira SC Zhu Y Splitter GA. Recombinant L7/L12 ribosomal protein and gamma-irradiated *Brucella abortus* induce a T-helper 1 subset response from murine CD4+ T cells. Immunology. (1994) 83:659–64. 7875746 PMC1415073

[20] Oliveira SC Splitter GA. Immunization of mice with recombinant L7/L12 ribosomal protein confers protection against *Brucella abortus* infection. Vaccine. (1996) 14:959–62. doi: 10.1016/0264-410x(96)00018-7, PMID: 8873388

[21] Jafari R Zolbanin NM Rafatpanah H Majidi J Kazemi T. Fc-fusion proteins in therapy: an updated view. Curr Med Chem. (2017) 24:1228–37. doi: 10.2174/0929867324666170113112759, PMID: 28088904

[22] Kontermann RE. Strategies for extended serum half-life of protein therapeutics. Curr Opin Biotechnol. (2011) 22:868–76. doi: 10.1016/j.copbio.2011.06.012 21862310

[23] Ye L Zeng R Bai Y Roopenian DC Zhu X. Efficient mucosal vaccination mediated by the neonatal fc receptor. Nat Biotechnol. (2011) 29:158–63. doi: 10.1038/nbt.1742, PMID: 21240266 PMC3197702

[24] Alleva DG Delpero AR Scully MM Murikipudi S Ragupathy R Greaves EK. Development of an IgG-fc fusion COVID-19 subunit vaccine, AKS-452. Vaccine. (2021) 39:6601–13. doi: 10.1016/j.vaccine.2021.09.077, PMID: 34642088 PMC8491978

[25] Doytchinova IA Flower DR. VaxiJen: a server for prediction of protective antigens, tumour antigens and subunit vaccines. BMC Bioinfo. (2007) 8:4. doi: 10.1186/1471-2105-8-4, PMID: 17207271 PMC1780059

[26] Kim Y Ponomarenko J Zhu Z Tamang D Wang P Greenbaum J. Immune epitope database analysis resource. Nucleic Acids Res. (2012) 40:W525–30. doi: 10.1093/nar/gks438 22610854 PMC3394288

[27] Zhang Q Wang P Kim Y Haste-Andersen P Beaver J Bourne PE. Immune epitope database analysis resource (IEDB-AR). Nucleic Acids Res. (2008) 36:W513–8. doi: 10.1093/nar/gkn254 18515843 PMC2447801

[28] Dhanda SK Vir P Raghava GPS. Designing of interferon-gamma inducing MHC class-II binders. Biol Direct. (2013) 8:30. doi: 10.1186/1745-6150-8-30, PMID: 24304645 PMC4235049

[29] Saha S Raghava GPS. Prediction of continuous B-cell epitopes in an antigen using recurrent neural network. Proteins: Struc, Func Bioinfo. (2006) 65:40–8. doi: 10.1002/prot.21078 16894596

[30] Cuspoca AF Díaz LL Acosta AF Peñaloza MK Méndez YR Clavijo DC. An Immunoinformatics approach for SARS-CoV-2 in Latam populations and multi-epitope vaccine candidate directed towards the World’s population. Vaccine. (2021) 9:581. doi: 10.3390/vaccines9060581, PMID: 34205992 PMC8228945

[31] Magnan CN Randall A Baldi P. SOLpro: accurate sequence-based prediction of protein solubility. Bioinformatics. (2009) 25:2200–7. doi: 10.1093/bioinformatics/btp386, PMID: 19549632

[32] Saha S Raghava GPS. AlgPred: prediction of allergenic proteins and mapping of IgE epitopes. Nucleic Acids Res. (2006) 34:W202–9. doi: 10.1093/nar/gkl343 16844994 PMC1538830

[33] Gupta S Kapoor P Chaudhary K Gautam A Kumar R Null N. In silico approach for predicting toxicity of peptides and proteins. PLoS One. (2013) 34:W202–9. doi: 10.1371/journal.pone.0073957 PMC3772798 24058508

[34] Baek M DiMaio F Anishchenko I Dauparas J Ovchinnikov S Lee GR. Accurate prediction of protein structures and interactions using a three-track neural network. Science. (2021) 373:871–6. doi: 10.1126/science.abj8754, PMID: 34282049 PMC7612213

[35] Wiederstein M Sippl MJ. ProSA-web: interactive web service for the recognition of errors in three-dimensional structures of proteins. Nucleic Acids Res. (2007) 35:W407–10. doi: 10.1093/nar/gkm290 17517781 PMC1933241

[36] Laskowski RA MacArthur MW Moss DS Thornton JM. PROCHECK: a program to check the stereochemical quality of protein structures. J Appl Crystallogr. (1993) 26:283–91. doi: 10.1107/s0021889892009944

[37] Colovos C Yeates TO. Verification of protein structures: patterns of nonbonded atomic interactions. Protein Sci. (1993) 2:1511–9. doi: 10.1002/pro.5560020916, PMID: 8401235 PMC2142462

[38] Alghamdi W Attique M Alzahrani E Ullah MZ Khan YD. LBCEPred: a machine learning model to predict linear B-cell epitopes. Brief Bioinform. (2022) 23:bbac035. doi: 10.1093/bib/bbac035 35262658

[39] Ponomarenko J Bui H-H Li W Fusseder N Bourne PE Sette A. ElliPro: a new structure-based tool for the prediction of antibody epitopes. BMC Bioinfo. (2008) 23:bbac035. doi: 10.1186/1471-2105-9-514 PMC2607291 19055730

[40] Cheng P Wang L Gong W. In silico analysis of peptide-based biomarkers for the diagnosis and prevention of latent tuberculosis infection. Front Microbiol. (2022) 13:947852. doi: 10.3389/fmicb.2022.947852, PMID: 35836423 PMC9273951

[41] Kozakov D Hall DR Xia B Porter KA Padhorny D Yueh C. The ClusPro web server for protein-protein docking. Nat Protoc. (2017) 12:255–78. doi: 10.1038/nprot.2016.169, PMID: 28079879 PMC5540229

[42] Krissinel E Henrick K. Inference of macromolecular assemblies from crystalline state. J Mol Biol. (2007) 372:774–97. doi: 10.1016/j.jmb.2007.05.022 17681537

[43] Lopéz-Blanco JR Garzón JI Chacón P. iMod: multipurpose normal mode analysis in internal coordinates. Bioinformatics. (2011) 27:2843–50. doi: 10.1093/bioinformatics/btr497, PMID: 21873636

[44] Rapin N Lund O Bernaschi M Castiglione F. Computational immunology meets bioinformatics: the use of prediction tools for molecular binding in the simulation of the immune system. PLoS One. (2010) 5:e9862. doi: 10.1371/journal.pone.0009862, PMID: 20419125 PMC2855701

[45] Castiglione F Mantile F De Berardinis P Prisco A. How the interval between prime and boost injection affects the immune response in a computational model of the immune system. Comput Math Methods Med. (2012) 2012:842329. doi: 10.1155/2012/842329, PMID: 22997539 PMC3446774

[46] Grote A Hiller K Scheer M Munch R Nortemann B Hempel DC. JCat: a novel tool to adapt codon usage of a target gene to its potential expression host. Nucleic Acids Res. (2005) 33:W526–31. doi: 10.1093/nar/gki376 15980527 PMC1160137

[47] Qian W Yang J-R Pearson NM Maclean C Zhang J. Balanced codon usage optimizes eukaryotic translational efficiency. PLoS Genet. (2012) 8:e1002603. doi: 10.1371/journal.pgen.1002603 22479199 PMC3315465

[48] Yin D Bai Q Li L Xu K Zhang J. Study on immunogenicity and antigenicity of a novel brucella multiepitope recombined protein. Biochem Biophys Res Commun. (2021) 540:37–41. doi: 10.1016/j.bbrc.2020.12.098, PMID: 33429198

[49] Greenwood B. The contribution of vaccination to global health: past, present and future. Philosop Trans Royal Society B: Biolog Sci. (2014) 369:20130433. doi: 10.1098/rstb.2013.0433, PMID: 24821919 PMC4024226

[50] Ficht TA Kahl-McDonagh MM Arenas-Gamboa AM. Rice-Ficht AC Brucellosis: the case for live, attenuated vaccines, 27. Vaccine. (2009) 27:D40–3. doi: 10.1016/j.vaccine.2009.08.058, PMID: PMC2780424 19837284

[51] Hou H Liu X Peng Q. The advances in brucellosis vaccines. Vaccine. (2019) 37:3981–8. doi: 10.1016/j.vaccine.2019.05.084, PMID: 31176541

[52] Moyle PM Toth I. Modern subunit vaccines: development, components, and research opportunities. ChemMedChem. (2013) 8:360–76. doi: 10.1002/cmdc.201200487 23316023

[53] Kardani K Bolhassani A Namvar A. An overview of in silico vaccine design against different pathogens and cancer. Expert Rev Vaccines. (2020) 19:699–726. doi: 10.1080/14760584.2020.1794832, PMID: 32648830

[54] Raoufi E Hemmati M Eftekhari S Khaksaran K Mahmodi Z Farajollahi MM. Epitope prediction by novel Immunoinformatics approach: a state-of-the-art review. Int J Peptide Res Therapeutics. (2019) 26:1155–63. doi: 10.1007/s10989-019-09918-z PMC7224030 32435171

[55] Detilleux PG Deyoe BL Cheville NF. Entry and intracellular localization of Brucella spp. in Vero cells: fluorescence and electron microscopy. Vet Pathol. (1990) 27:317–28. doi: 10.1177/030098589002700503, PMID: 2122572

[56] de Figueiredo P Ficht TA Rice-Ficht A Rossetti CA Adams LG. Pathogenesis and immunobiology of brucellosis: review of Brucella-host interactions. Am J Pathol. (2015) 185:1505–17. doi: 10.1016/j.ajpath.2015.03.003, PMID: 25892682 PMC4450313

[57] Goenka R Guirnalda PD Black SJ Baldwin CL. B lymphocytes provide an infection niche for intracellular bacterium *Brucella abortus*. J Infect Dis. (2012) 206:91–8. doi: 10.1093/infdis/jis310, PMID: 22561364 PMC3415929

[58] Goenka R Parent MA Elzer PH Baldwin CL. B cell-deficient mice display markedly enhanced resistance to the intracellular bacterium *Brucella abortus*. J Infect Dis. (2011) 203:1136–46. doi: 10.1093/infdis/jiq171 21451002

[59] Rawal K Sinha R Abbasi BA Chaudhary A Nath SK Kumari P. Identification of vaccine targets in pathogens and design of a vaccine using computational approaches. Sci Rep. (2021) 11:17626. doi: 10.1038/s41598-021-96863-x 34475453 PMC8413327

[60] Sanches RCO Tiwari S Ferreira LCG Oliveira FM Lopes MD Passos MJF. Immunoinformatics design of multi-epitope peptide-based vaccine against *Schistosoma mansoni* using transmembrane proteins as a target. Front Immunol. (2021) 12:621706. doi: 10.3389/fimmu.2021.621706, PMID: 33737928 PMC7961083

[61] Loureiro S Ren J Phapugrangkul P Colaco CA Bailey CR Shelton H. Adjuvant-free immunization with hemagglutinin-fc fusion proteins as an approach to influenza vaccines. J Virol. (2010) 85:3010–4. doi: 10.1128/jvi.01241-10, PMID: 21191017 PMC3067967

[62] Soleimanpour S Farsiani H Mosavat A Ghazvini K Eydgahi MRA Sankian M. APC targeting enhances immunogenicity of a novel multistage fc-fusion tuberculosis vaccine in mice. Appl Microbiol Biotechnol. (2015) 99:10467–80. doi: 10.1007/s00253-015-6952-z, PMID: 26373723

[63] Li J Li X Ma H Ren X Hao G Zhang H. Efficient mucosal vaccination of a novel classical swine fever virus E2-fc fusion protein mediated by neonatal fc receptor. Vaccine. (2020) 38:4574–83. doi: 10.1016/j.vaccine.2020.05.013, PMID: 32417139

[64] Meza B Ascencio F Sierra-Beltrán AP Torres J Angulo C. A novel design of a multi-antigenic, multistage and multi-epitope vaccine against *Helicobacter pylori*: an in silico approach. Infect Genet Evol. (2017) 49:309–17. doi: 10.1016/j.meegid.2017.02.007 28185986

[65] García-Valtanen P Martinez-Lopez A Ortega-Villaizan M Perez L Coll JM Estepa A. In addition to its antiviral and immunomodulatory properties, the zebrafish β-defensin 2 (zfBD2) is a potent viral DNA vaccine molecular adjuvant. Antivir Res. (2013) 101:136–47. doi: 10.1016/j.antiviral.2013.11.009, PMID: 24286781

[66] Solanki V Tiwari V. Subtractive proteomics to identify novel drug targets and reverse vaccinology for the development of chimeric vaccine against *Acinetobacter baumannii*. Sci Rep. (2018) 8:9044. doi: 10.1038/s41598-018-26689-7 29899345 PMC5997985

[67] Barh D Barve N Gupta K Chandra S Jain N Tiwari S. Exoproteome and secretome derived broad spectrum novel drug and vaccine candidates in *Vibrio cholerae* targeted by *Piper betel* derived compounds. PLoS One. (2013) 8:e52773. doi: 10.1371/journal.pone.0052773, PMID: 23382822 PMC3559646

[68] Yu M Zhu Y Li Y Chen Z Li Z Wang J. Design of a Recombinant Multivalent Epitope Vaccine Based on SARS-CoV-2 and its variants in Immunoinformatics approaches. Front Immunol. (2022) 13:884433. doi: 10.3389/fimmu.2022.884433, PMID: 35603198 PMC9120605

[69] Dominguez-Flores A Rodríguez López GM Soria-Castro R López-Santiago R Rodríguez-Cortés O Pérez-Tapia SM. *Brucella abortus* induces mast cell activation through TLR-2 and TLR-4. Microb Pathog. (2023) 176:106005. doi: 10.1016/j.micpath.2023.106005, PMID: 36717005

[70] Li J-Y Liu Y Gao X-X Gao X Cai H. TLR2 and TLR4 signaling pathways are required for recombinant *Brucella abortus* BCSP31-induced cytokine production, functional upregulation of mouse macrophages, and the Th1 immune response in vivo and in vitro. Cell Mol Immunol. (2023) 11:477–94. doi: 10.1038/cmi.2014.28, PMID: 24769793 PMC4197203

[71] Skendros P Pappas G Boura P. Cell-mediated immunity in human brucellosis. Microbes Infect. (2010) 13:134–42. doi: 10.1016/j.micinf.2010.10.015 21034846

[72] Mezouar S Mege J-L. Changing the paradigm of IFN-γ at the interface between innate and adaptive immunity: macrophage-derived IFN-γ. J Leukoc Biol. (2020) 108:419–26. doi: 10.1002/jlb.4mir0420-619rr, PMID: 32531848

