# Supplementary material for: Design of a multi-epitope vaccine against brucellosis fused to IgG-fc by an immunoinformatics approach
Source: Front Vet Sci. 2023 Oct 23;10:1238634. doi: 10.3389/fvets.2023.1238634 (PMC10625910; doi:10.3389/fvets.2023.1238634)
Supplement: Supplementary file 1 [file Data_Sheet_1.docx]

Supplementary Material

**Design of a multi-epitope vaccine against brucellosis fused to IgG-Fc by an immunoinformatics approach**

Aodi Wu ^1^, Yueli Wang^1^, Adnan Ali^1^ , Zhenyu Xu^1^, Dongsheng Zhang^1^, Kairat zhumanov^2^, Jihai Yi^2*^, Jinliang Sheng^1*^

*** Correspondence:**

Jinliang Sheng 1572621211@qq.com

Jihai Yi 724050645@qq.com

# Supplementary Figures and Tables

## Supplementary Figures


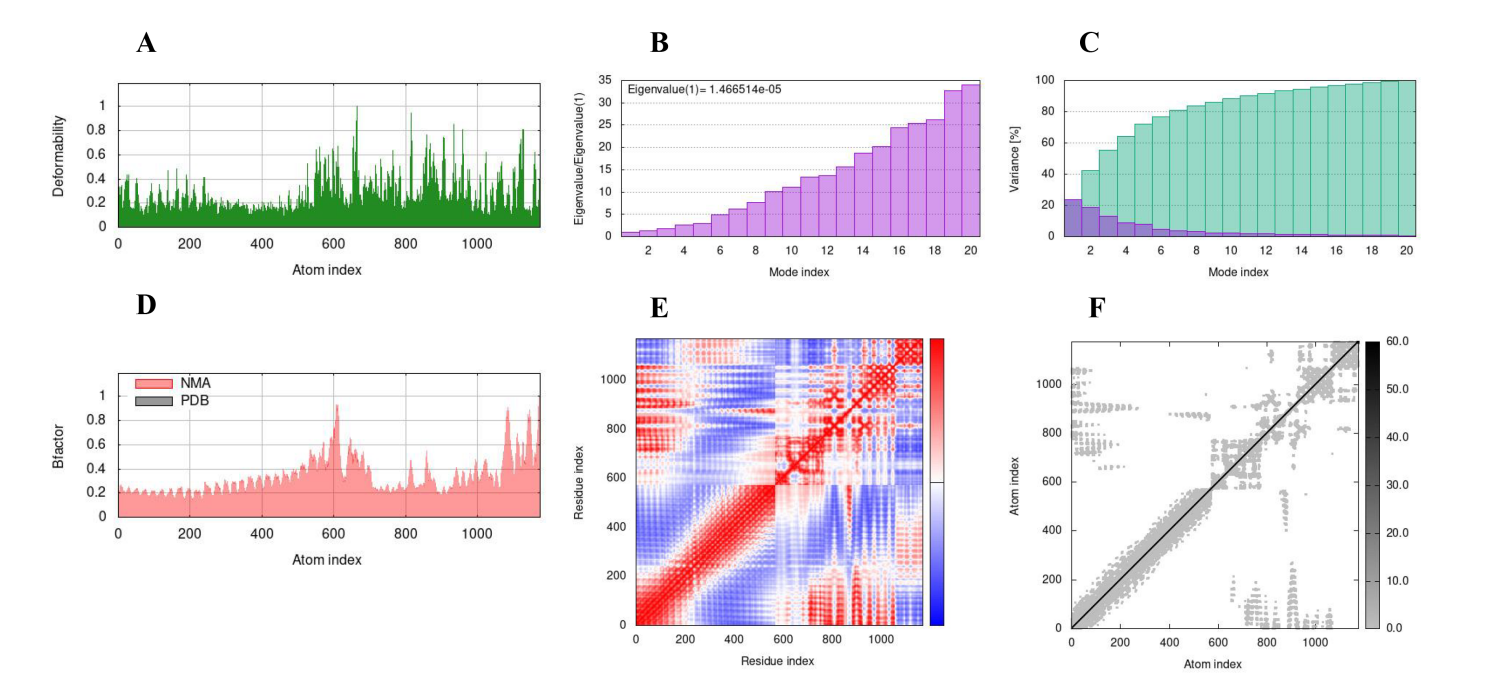


**Supplementary Figure1.** Molecular dynamics simulation results of MEV-Fc-TLR4.

## Supplementary Tables

**Supplementary Table 1.** **Candidate CTL epitopes with percentile rank < 0.5**

| antigen | allele | peptide | length | percentile  rank |
| --- | --- | --- | --- | --- |
| L7/L12 | HLA-A*26:01 | NVIKEVRAL | 9 | 0.14 |
|  | HLA-B*08:01 | NVIKEVRAL | 9 | 0.23 |
|  | HLA-A*68:02 | NVIKEVRAL | 9 | 0.28 |
|  | HLA-A*02:06 | NVIKEVRAL | 9 | 0.46 |
|  | HLA-A*31:01 | KINVIKEVR | 9 | 0.06 |
|  | HLA-A*11:01 | GANKINVIK | 9 | 0.23 |
|  | HLA-A*30:01 | GANKINVIK | 9 | 0.26 |
|  | HLA-A*02:06 | KIVEDLSAL | 9 | 0.06 |
|  | HLA-A*02:03 | KIVEDLSAL | 9 | 0.11 |
|  | HLA-A*02:01 | KIVEDLSAL | 9 | 0.17 |
|  | HLA-A*11:01 | TVLEAAELSK | 10 | 0.12 |
|  | HLA-A*03:01 | TVLEAAELSK | 10 | 0.34 |
|  | HLA-A*68:02 | EAKDLVEGA | 9 | 0.29 |
|  | HLA-B*40:01 | KEVRALTGL | 9 | 0.06 |
|  | HLA-B*44:03 | KEVRALTGL | 9 | 0.47 |
|  | HLA-B*35:01 | AAAEEKTEF | 9 | 0.19 |
|  | HLA-B*15:01 | AAAEEKTEF | 9 | 0.42 |
|  | HLA-A*68:02 | AAAPVAVAA | 9 | 0.38 |
|  | HLA-A*68:02 | SAAAPVAVA | 9 | 0.43 |
|  | HLA-A*02:01 | SKLLEEKWGV | 10 | 0.13 |
|  | HLA-A*02:06 | SKLLEEKWGV | 10 | 0.41 |
|  | HLA-A*68:02 | VSAAAPVAV | 9 | 0.42 |
|  | HLA-A*02:01 | KLLEEKWGV | 9 | 0.01 |
|  | HLA-A*02:06 | KLLEEKWGV | 9 | 0.02 |
|  | HLA-A*02:03 | KLLEEKWGV | 9 | 0.08 |
|  | HLA-A*32:01 | KLLEEKWGV | 9 | 0.4 |
|  | HLA-A*02:03 | VLADGGANKI | 10 | 0.45 |
|  | HLA-A*11:01 | VVLADGGANK | 10 | 0.22 |
|  | HLA-A*03:01 | VVLADGGANK | 10 | 0.3 |
|  | HLA-A*30:01 | RALTGLGLK | 9 | 0.1 |
|  | HLA-A*03:01 | RALTGLGLK | 9 | 0.26 |
|  | HLA-A*11:01 | RALTGLGLK | 9 | 0.29 |
|  | HLA-B*40:01 | EEKTEFDVVL | 10 | 0.39 |
|  | HLA-A*03:01 | VLADGGANK | 9 | 0.12 |
|  | HLA-A*11:01 | VLADGGANK | 9 | 0.31 |
|  | HLA-A*02:06 | AQLEAAGAKV | 10 | 0.39 |
|  | HLA-B*40:01 | LEAAGAKVEL | 10 | 0.12 |
|  | HLA-A*68:01 | EAAGAKVELK | 10 | 0.07 |
|  | HLA-A*68:02 | EAAGAKVEL | 9 | 0.08 |
|  | HLA-B*35:01 | EAAGAKVEL | 9 | 0.39 |
|  | HLA-A*26:01 | EAAGAKVEL | 9 | 0.47 |
|  | HLA-B*57:01 | LSKLLEEKW | 9 | 0.01 |
|  | HLA-B*58:01 | LSKLLEEKW | 9 | 0.01 |
|  | HLA-B*53:01 | LSKLLEEKW | 9 | 0.35 |
|  | HLA-A*11:01 | AAGAKVELK | 9 | 0.35 |
|  | HLA-A*30:01 | KIKAQLEAA | 9 | 0.4 |
|  | HLA-B*40:01 | VLEAAELSKL | 10 | 0.39 |
|  | HLA-B*40:01 | VEDLSALTVL | 10 | 0.11 |
|  | HLA-B*07:02 | APKAVKEGA | 9 | 0.45 |
|  | HLA-B*40:01 | VEDLSALTV | 9 | 0.2 |
|  | HLA-A*02:03 | GLKEAKDLV | 9 | 0.21 |
|  | HLA-B*40:01 | LEAAELSKL | 9 | 0.07 |
|  | HLA-B*44:03 | LEAAELSKL | 9 | 0.48 |
|  | HLA-A*30:01 | KAVKEGASK | 9 | 0.26 |
|  | HLA-A*11:01 | KAVKEGASK | 9 | 0.44 |
|  | HLA-A*68:01 | ELSKLLEEK | 9 | 0.38 |
|  | HLA-B*44:03 | DEAEKIKAQL | 10 | 0.41 |
|  | HLA-B*44:02 | DEAEKIKAQL | 10 | 0.41 |
|  | HLA-B*40:01 | LEAAELSKLL | 10 | 0.28 |
|  | HLA-B*57:01 | ELSKLLEEKW | 10 | 0.34 |
|  | HLA-A*68:02 | EAAELSKLL | 9 | 0.06 |
|  | HLA-B*51:01 | EAAELSKLL | 9 | 0.3 |
|  | HLA-A*26:01 | EAAELSKLL | 9 | 0.34 |
|  | HLA-B*53:01 | EAAELSKLL | 9 | 0.41 |
|  | HLA-A*68:02 | EAEKIKAQL | 9 | 0.46 |
| OMP16 | HLA-B*58:01 | AVCDADTCW | 9 | 0.21 |
|  | HLA-B*57:01 | AVCDADTCW | 9 | 0.37 |
|  | HLA-B*58:01 | VAVCDADTCW | 10 | 0.25 |
|  | HLA-B*53:01 | VAVCDADTCW | 10 | 0.39 |
|  | HLA-B*57:01 | VAVCDADTCW | 10 | 0.45 |
|  | HLA-B*15:01 | KQAQWLQRY | 9 | 0.01 |
|  | HLA-A*30:02 | KQAQWLQRY | 9 | 0.01 |
|  | HLA-A*32:01 | KQAQWLQRY | 9 | 0.05 |
|  | HLA-A*26:01 | KQAQWLQRY | 9 | 0.47 |
|  | HLA-B*44:03 | KQAQWLQRY | 9 | 0.5 |
|  | HLA-B*15:01 | SKQAQWLQRY | 10 | 0.17 |
|  | HLA-A*30:02 | SKQAQWLQRY | 10 | 0.17 |
|  | HLA-A*31:01 | ATRDFLASR | 9 | 0.01 |
|  | HLA-A*30:01 | ATRDFLASR | 9 | 0.07 |
|  | HLA-A*33:01 | ATRDFLASR | 9 | 0.08 |
|  | HLA-A*11:01 | ATRDFLASR | 9 | 0.16 |
|  | HLA-A*68:01 | ATRDFLASR | 9 | 0.2 |
|  | HLA-A*03:01 | ATRDFLASR | 9 | 0.29 |
|  | HLA-A*68:01 | MRTISYGNER | 10 | 0.15 |
|  | HLA-B*07:02 | LPNNAGDLGL | 10 | 0.32 |
|  | HLA-A*68:02 | FTVNVGDRI | 9 | 0.29 |
|  | HLA-A*68:01 | VTVLNGAGR | 9 | 0.26 |
|  | HLA-A*31:01 | AATRDFLASR | 10 | 0.05 |
|  | HLA-A*33:01 | AATRDFLASR | 10 | 0.45 |
|  | HLA-B*07:02 | IARSPIAIAL | 10 | 0.35 |
|  | HLA-B*58:01 | RSPIAIALF | 9 | 0.32 |
|  | HLA-A*24:02 | RSPIAIALF | 9 | 0.32 |
|  | HLA-A*23:01 | RSPIAIALF | 9 | 0.34 |
|  | HLA-B*57:01 | RSPIAIALF | 9 | 0.38 |
|  | HLA-A*32:01 | RSPIAIALF | 9 | 0.39 |
|  | HLA-A*68:01 | LASRGVPTNR | 10 | 0.41 |
|  | HLA-B*08:01 | YGNERPVAV | 9 | 0.07 |
|  | HLA-B*51:01 | YGNERPVAV | 9 | 0.5 |
|  | HLA-A*31:01 | ASRGVPTNR | 9 | 0.01 |
|  | HLA-A*30:01 | ASRGVPTNR | 9 | 0.07 |
|  | HLA-B*08:01 | SQNRRAVTV | 9 | 0.17 |
|  | HLA-A*02:06 | SQNRRAVTV | 9 | 0.36 |
|  | HLA-A*02:03 | SQNRRAVTV | 9 | 0.37 |
|  | HLA-B*35:01 | SPIAIALFM | 9 | 0.09 |
|  | HLA-B*53:01 | SPIAIALFM | 9 | 0.13 |
|  | HLA-B*07:02 | SPIAIALFM | 9 | 0.3 |
|  | HLA-B*51:01 | SPIAIALFM | 9 | 0.41 |
|  | HLA-A*68:02 | SSQDFTVNV | 9 | 0.09 |
|  | HLA-A*02:06 | SSQDFTVNV | 9 | 0.15 |
|  | HLA-A*30:01 | GTREYNLAL | 9 | 0.31 |
|  | HLA-A*68:01 | ITIEGHADER | 10 | 0.22 |
|  | HLA-A*01:01 | HADERGTREY | 10 | 0.04 |
|  | HLA-B*35:01 | HADERGTREY | 10 | 0.12 |
|  | HLA-B*44:02 | ADERGTREY | 9 | 0.47 |
|  | HLA-A*02:03 | ALFMSLAVA | 9 | 0.31 |
|  | HLA-B*15:01 | AQWLQRYPQY | 10 | 0.21 |
|  | HLA-A*30:02 | AQWLQRYPQY | 10 | 0.24 |
|  | HLA-A*11:01 | AVAGCASKK | 9 | 0.1 |
|  | HLA-A*03:01 | AVAGCASKK | 9 | 0.12 |
|  | HLA-A*33:01 | DTCWSQNRR | 9 | 0.1 |
|  | HLA-A*68:01 | DTCWSQNRR | 9 | 0.2 |
|  | HLA-A*33:01 | EYNLALGQR | 9 | 0.05 |
|  | HLA-A*33:01 | EYNLALGQRR | 10 | 0.1 |
|  | HLA-A*02:03 | FLASRGVPT | 9 | 0.4 |
|  | HLA-B*51:01 | IAIALFMSL | 9 | 0.2 |
|  | HLA-B*51:01 | IALFMSLAV | 9 | 0.34 |
|  | HLA-A*23:01 | IFFDLDSSL | 9 | 0.37 |
|  | HLA-B*57:01 | QQTLSKQAQW | 10 | 0.04 |
|  | HLA-B*58:01 | QQTLSKQAQW | 10 | 0.08 |
|  | HLA-A*32:01 | QQTLSKQAQW | 10 | 0.32 |
|  | HLA-A*24:02 | QRYPQYSITI | 10 | 0.24 |
|  | HLA-A*23:01 | QRYPQYSITI | 10 | 0.39 |
|  | HLA-B*57:01 | QTLSKQAQW | 9 | 0.01 |
|  | HLA-B*58:01 | QTLSKQAQW | 9 | 0.01 |
|  | HLA-A*32:01 | QTLSKQAQW | 9 | 0.03 |
|  | HLA-B*53:01 | QTLSKQAQW | 9 | 0.24 |
|  | HLA-B*57:01 | QTLSKQAQWL | 10 | 0.5 |
|  | HLA-A*30:02 | QWLQRYPQY | 9 | 0.25 |
|  | HLA-A*11:01 | RADAQQTLSK | 10 | 0.25 |
|  | HLA-A*03:01 | RADAQQTLSK | 10 | 0.27 |
|  | HLA-A*30:01 | RADAQQTLSK | 10 | 0.31 |
|  | HLA-A*31:01 | RGVPTNRMR | 9 | 0.25 |
|  | HLA-A*31:01 | RTISYGNER | 9 | 0.02 |
|  | HLA-A*11:01 | RTISYGNER | 9 | 0.16 |
|  | HLA-A*68:01 | RTISYGNER | 9 | 0.3 |
|  | HLA-A*30:01 | RTISYGNER | 9 | 0.41 |
|  | HLA-A*24:02 | RYPQYSITI | 9 | 0.01 |
|  | HLA-A*23:01 | RYPQYSITI | 9 | 0.03 |
|  | HLA-A*30:02 | TNRMRTISY | 9 | 0.5 |
|  | HLA-B*51:01 | VPTNRMRTI | 9 | 0.05 |
|  | HLA-B*07:02 | VPTNRMRTI | 9 | 0.11 |
|  | HLA-B*08:01 | VPTNRMRTI | 9 | 0.32 |
| OMP19 | HLA-B*07:02 | GPLRCPGEL | 9 | 0.18 |
|  | HLA-A*68:01 | NAVPAGTVQK | 10 | 0.14 |
|  | HLA-B*53:01 | LTPGAVAGVW | 10 | 0.26 |
|  | HLA-B*57:01 | LTPGAVAGVW | 10 | 0.3 |
|  | HLA-B*58:01 | LTPGAVAGVW | 10 | 0.39 |
|  | HLA-A*02:03 | SLAAAGIVL | 9 | 0.47 |
|  | HLA-A*11:01 | AVPAGTVQK | 9 | 0.02 |
|  | HLA-A*03:01 | AVPAGTVQK | 9 | 0.1 |
|  | HLA-A*30:01 | AVPAGTVQK | 9 | 0.19 |
|  | HLA-B*53:01 | TPGAVAGVW | 9 | 0.03 |
|  | HLA-A*68:02 | LTPGAVAGV | 9 | 0.16 |
|  | HLA-A*02:03 | VLYDANGGTV | 10 | 0.29 |
|  | HLA-B*07:02 | APDLTPGAV | 9 | 0.2 |
|  | HLA-A*68:02 | DLTPGAVAGV | 10 | 0.32 |
|  | HLA-A*30:02 | ANGGTVASLY | 10 | 0.36 |
|  | HLA-A*11:01 | ASLGGQSCK | 9 | 0.1 |
|  | HLA-A*03:01 | ASLGGQSCK | 9 | 0.37 |
|  | HLA-A*30:01 | ASLGGQSCK | 9 | 0.42 |
|  | HLA-A*31:01 | ASLYSSGQGR | 10 | 0.46 |
|  | HLA-A*03:01 | ASLYSSGQGR | 10 | 0.47 |
|  | HLA-A*11:01 | ASLYSSGQGR | 10 | 0.48 |
|  | HLA-A*32:01 | AVNGKQLVL | 9 | 0.25 |
|  | HLA-B*08:01 | AVNGKQLVL | 9 | 0.26 |
|  | HLA-B*07:02 | AVNGKQLVL | 9 | 0.43 |
|  | HLA-A*30:02 | AVNGKQLVLY | 10 | 0.04 |
|  | HLA-B*15:01 | AVNGKQLVLY | 10 | 0.22 |
|  | HLA-A*01:01 | AVNGKQLVLY | 10 | 0.25 |
|  | HLA-A*26:01 | AVNGKQLVLY | 10 | 0.29 |
|  | HLA-A*03:01 | AVNGKQLVLY | 10 | 0.3 |
|  | HLA-A*11:01 | AVNGKQLVLY | 10 | 0.46 |
|  | HLA-A*30:02 | CKIATPQTKY | 10 | 0.41 |
|  | HLA-B*35:01 | FPNAPSTDM | 9 | 0.02 |
|  | HLA-B*07:02 | FPNAPSTDM | 9 | 0.07 |
|  | HLA-B*53:01 | FPNAPSTDM | 9 | 0.07 |
|  | HLA-B*51:01 | FPNAPSTDM | 9 | 0.31 |
|  | HLA-B*44:02 | GELANLASW | 9 | 0.01 |
|  | HLA-B*44:03 | GELANLASW | 9 | 0.02 |
|  | HLA-A*30:01 | ISKASLLSL | 9 | 0.15 |
|  | HLA-B*57:01 | ISKASLLSL | 9 | 0.41 |
|  | HLA-A*30:02 | KIATPQTKY | 9 | 0.01 |
|  | HLA-B*15:01 | KIATPQTKY | 9 | 0.03 |
|  | HLA-A*32:01 | KIATPQTKY | 9 | 0.13 |
|  | HLA-A*03:01 | KIATPQTKY | 9 | 0.14 |
|  | HLA-A*01:01 | KIATPQTKY | 9 | 0.25 |
|  | HLA-A*11:01 | KIATPQTKY | 9 | 0.31 |
|  | HLA-A*26:01 | KIATPQTKY | 9 | 0.31 |
|  | HLA-B*51:01 | LPPASAPDL | 9 | 0.3 |
|  | HLA-B*07:02 | LPPASAPDL | 9 | 0.47 |
|  | HLA-A*24:02 | LYSSGQGRF | 9 | 0.11 |
|  | HLA-A*23:01 | LYSSGQGRF | 9 | 0.14 |
|  | HLA-B*08:01 | MGISKASLL | 9 | 0.33 |
|  | HLA-A*68:02 | MSAQSGTQV | 9 | 0.22 |
|  | HLA-A*68:02 | NVSPPPPPA | 9 | 0.11 |
|  | HLA-B*07:02 | QFPNAPSTDM | 10 | 0.38 |
|  | HLA-A*68:01 | QTKYGQGYR | 9 | 0.28 |
|  | HLA-A*31:01 | QTKYGQGYR | 9 | 0.3 |
|  | HLA-A*33:01 | QTKYGQGYR | 9 | 0.31 |
|  | HLA-A*03:01 | SLYSSGQGR | 9 | 0.16 |
|  | HLA-A*31:01 | SLYSSGQGR | 9 | 0.19 |
|  | HLA-A*33:01 | SLYSSGQGR | 9 | 0.45 |
|  | HLA-A*11:01 | SLYSSGQGR | 9 | 0.47 |
|  | HLA-B*15:01 | SLYSSGQGRF | 10 | 0.12 |
|  | HLA-A*24:02 | SLYSSGQGRF | 10 | 0.38 |
|  | HLA-B*07:02 | SPPPPPAPV | 9 | 0.04 |
|  | HLA-B*51:01 | SPPPPPAPV | 9 | 0.05 |
|  | HLA-A*68:02 | SPPPPPAPV | 9 | 0.38 |
|  | HLA-B*35:01 | TPQTKYGQGY | 10 | 0.4 |
|  | HLA-B*15:01 | TPQTKYGQGY | 10 | 0.47 |
|  | HLA-A*02:06 | TQVASLPPA | 9 | 0.43 |
|  | HLA-A*30:02 | VNGKQLVLY | 9 | 0.41 |
|  | HLA-B*51:01 | VSPPPPPAPV | 10 | 0.35 |
|  | HLA-B*07:02 | VSPPPPPAPV | 10 | 0.45 |
| OMP25 | HLA-B*07:02 | KPNGFIGGL | 9 | 0.06 |
|  | HLA-A*30:02 | AYFGGQVGY | 9 | 0.01 |
|  | HLA-A*30:02 | GAYFGGQVGY | 10 | 0.09 |
|  | HLA-B*15:01 | GAYFGGQVGY | 10 | 0.14 |
|  | HLA-A*24:02 | PYIAGGVAF | 9 | 0.06 |
|  | HLA-A*23:01 | PYIAGGVAF | 9 | 0.08 |
|  | HLA-B*35:01 | MPYIAGGVAF | 10 | 0.01 |
|  | HLA-B*53:01 | MPYIAGGVAF | 10 | 0.04 |
|  | HLA-B*07:02 | MPYIAGGVAF | 10 | 0.08 |
|  | HLA-A*24:02 | MPYIAGGVAF | 10 | 0.18 |
|  | HLA-A*23:01 | MPYIAGGVAF | 10 | 0.18 |
|  | HLA-B*51:01 | MPYIAGGVAF | 10 | 0.23 |
|  | HLA-A*24:02 | SFSWAGAYF | 9 | 0.14 |
|  | HLA-A*23:01 | SFSWAGAYF | 9 | 0.14 |
|  | HLA-B*35:01 | YAIDRFMPY | 9 | 0.02 |
|  | HLA-A*26:01 | YAIDRFMPY | 9 | 0.02 |
|  | HLA-B*15:01 | YAIDRFMPY | 9 | 0.13 |
|  | HLA-A*30:02 | YAIDRFMPY | 9 | 0.16 |
|  | HLA-B*40:01 | IEYAATDNVL | 10 | 0.12 |
|  | HLA-A*03:01 | QVGYGWGRAK | 10 | 0.21 |
|  | HLA-A*31:01 | RWSGAVRAR | 9 | 0.27 |
|  | HLA-A*23:01 | GYNFDTGNNF | 10 | 0.09 |
|  | HLA-A*24:02 | GYNFDTGNNF | 10 | 0.11 |
|  | HLA-A*68:01 | YAATDNVLLR | 10 | 0.16 |
|  | HLA-A*33:01 | NVLLRLEYR | 9 | 0.02 |
|  | HLA-A*68:01 | NVLLRLEYR | 9 | 0.34 |
|  | HLA-A*31:01 | NVLLRLEYR | 9 | 0.35 |
|  | HLA-A*01:01 | ATDNVLLRL | 9 | 0.19 |
|  | HLA-A*02:06 | ATDNVLLRL | 9 | 0.24 |
|  | HLA-A*68:01 | AATDNVLLR | 9 | 0.22 |
|  | HLA-A*11:01 | AATDNVLLR | 9 | 0.33 |
|  | HLA-A*33:01 | DNVLLRLEYR | 10 | 0.13 |
|  | HLA-A*26:01 | WTVGAGIEY | 9 | 0.05 |
|  | HLA-A*01:01 | WTVGAGIEY | 9 | 0.14 |
|  | HLA-B*35:01 | WTVGAGIEY | 9 | 0.17 |
|  | HLA-A*30:02 | VLLRLEYRY | 9 | 0.13 |
|  | HLA-B*35:01 | APIAIAPSF | 9 | 0.02 |
|  | HLA-B*53:01 | APIAIAPSF | 9 | 0.03 |
|  | HLA-B*07:02 | APIAIAPSF | 9 | 0.04 |
|  | HLA-B*51:01 | APIAIAPSF | 9 | 0.19 |
|  | HLA-A*68:01 | DIRLGVAYK | 9 | 0.48 |
|  | HLA-A*68:02 | FKTNDIRLGV | 10 | 0.19 |
|  | HLA-B*58:01 | PIAIAPSFSW | 10 | 0.29 |
|  | HLA-B*57:01 | PIAIAPSFSW | 10 | 0.3 |
|  | HLA-A*30:01 | KTNDIRLGV | 9 | 0.06 |
|  | HLA-A*02:06 | KTNDIRLGV | 9 | 0.18 |
|  | HLA-A*68:02 | KTNDIRLGV | 9 | 0.19 |
|  | HLA-A*32:01 | RTNGGTSEF | 9 | 0.02 |
|  | HLA-B*15:01 | RTNGGTSEF | 9 | 0.08 |
|  | HLA-A*30:02 | RTNGGTSEF | 9 | 0.14 |
|  | HLA-B*58:01 | RTNGGTSEF | 9 | 0.15 |
|  | HLA-B*57:01 | RTNGGTSEF | 9 | 0.34 |
|  | HLA-B*57:01 | QTTGETQLRW | 10 | 0.04 |
|  | HLA-B*58:01 | QTTGETQLRW | 10 | 0.04 |
|  | HLA-B*58:01 | TTGETQLRW | 9 | 0.01 |
|  | HLA-B*57:01 | TTGETQLRW | 9 | 0.03 |
|  | HLA-A*02:06 | FILGLDANV | 9 | 0.17 |
|  | HLA-A*02:01 | FILGLDANV | 9 | 0.28 |
|  | HLA-A*68:01 | QTTGETQLR | 9 | 0.07 |
|  | HLA-A*03:01 | RTNGGTSEFK | 10 | 0.05 |
|  | HLA-A*11:01 | RTNGGTSEFK | 10 | 0.07 |
|  | HLA-A*30:01 | RTNGGTSEFK | 10 | 0.11 |
|  | HLA-B*15:01 | AVITSTSAY | 9 | 0.01 |
|  | HLA-A*30:02 | AVITSTSAY | 9 | 0.01 |
|  | HLA-A*26:01 | AVITSTSAY | 9 | 0.03 |
|  | HLA-B*35:01 | AVITSTSAY | 9 | 0.12 |
|  | HLA-A*11:01 | AVITSTSAY | 9 | 0.29 |
|  | HLA-A*33:01 | DYNNLKKSR | 9 | 0.02 |
|  | HLA-B*58:01 | FGGQVGYGW | 9 | 0.25 |
|  | HLA-B*57:01 | FGGQVGYGW | 9 | 0.39 |
|  | HLA-B*58:01 | IAIAPSFSW | 9 | 0.01 |
|  | HLA-B*57:01 | IAIAPSFSW | 9 | 0.01 |
|  | HLA-B*53:01 | IAIAPSFSW | 9 | 0.02 |
|  | HLA-A*32:01 | IAIAPSFSW | 9 | 0.05 |
|  | HLA-A*02:01 | LLGASLVAV | 9 | 0.14 |
|  | HLA-A*02:03 | LLGASLVAV | 9 | 0.14 |
|  | HLA-A*68:02 | MTFKNLLGA | 9 | 0.14 |
|  | HLA-A*24:02 | RYTDYGKKNF | 10 | 0.14 |
|  | HLA-A*23:01 | RYTDYGKKNF | 10 | 0.15 |
|  | HLA-B*40:01 | SEFKPNGFI | 9 | 0.15 |
|  | HLA-B*57:01 | SSKSKTQTGW | 10 | 0.04 |
|  | HLA-B*58:01 | SSKSKTQTGW | 10 | 0.07 |
|  | HLA-B*15:01 | VAVITSTSAY | 10 | 0.16 |
|  | HLA-A*01:01 | YTDYGKKNF | 9 | 0.22 |

**Supplementary Table 2.HTL epitopes with percentile rank < 0.5**

| antigen | allele | peptide | length | Percentile  rank |
| --- | --- | --- | --- | --- |
| L7L12 | HLA-DQA1*05:01/DQB1*03:01 | AAAGGAAPAAAAEEK | 15 | 0.01 |
|  | HLA-DQA1*05:01/DQB1*03:01 | APVAVAAAGGAAPAA | 15 | 0.01 |
|  | HLA-DQA1*05:01/DQB1*03:01 | AVAAAGGAAPAAAAE | 15 | 0.01 |
|  | HLA-DQA1*05:01/DQB1*03:01 | PVAVAAAGGAAPAAA | 15 | 0.01 |
|  | HLA-DQA1*05:01/DQB1*03:01 | VAAAGGAAPAAAAEE | 15 | 0.01 |
|  | HLA-DQA1*05:01/DQB1*03:01 | VAVAAAGGAAPAAAA | 15 | 0.01 |
|  | HLA-DQA1*05:01/DQB1*03:01 | AAGGAAPAAAAEEKT | 15 | 0.02 |
|  | HLA-DQA1*05:01/DQB1*03:01 | AAPVAVAAAGGAAPA | 15 | 0.02 |
|  | HLA-DQA1*05:01/DQB1*03:01 | AAAPVAVAAAGGAAP | 15 | 0.03 |
|  | HLA-DQA1*01:02/DQB1*06:02 | IVEDLSALTVLEAAE | 15 | 0.04 |
|  | HLA-DQA1*01:02/DQB1*06:02 | VEDLSALTVLEAAEL | 15 | 0.04 |
|  | HLA-DQA1*01:02/DQB1*06:02 | EDLSALTVLEAAELS | 15 | 0.06 |
|  | HLA-DQA1*05:01/DQB1*03:01 | KWGVSAAAPVAVAAA | 15 | 0.09 |
|  | HLA-DQA1*05:01/DQB1*03:01 | WGVSAAAPVAVAAAG | 15 | 0.09 |
|  | HLA-DQA1*05:01/DQB1*03:01 | EKWGVSAAAPVAVAA | 15 | 0.12 |
|  | HLA-DQA1*05:01/DQB1*03:01 | GVSAAAPVAVAAAGG | 15 | 0.15 |
|  | HLA-DQA1*05:01/DQB1*03:01 | SAAAPVAVAAAGGAA | 15 | 0.15 |
|  | HLA-DQA1*05:01/DQB1*03:01 | EEKWGVSAAAPVAVA | 15 | 0.17 |
|  | HLA-DQA1*01:02/DQB1*06:02 | KIVEDLSALTVLEAA | 15 | 0.19 |
|  | HLA-DRB1*09:01 | EEKWGVSAAAPVAVA | 15 | 0.21 |
|  | HLA-DQA1*05:01/DQB1*03:01 | VSAAAPVAVAAAGGA | 15 | 0.21 |
|  | HLA-DRB1*09:01 | EKWGVSAAAPVAVAA | 15 | 0.22 |
|  | HLA-DRB1*09:01 | KWGVSAAAPVAVAAA | 15 | 0.33 |
|  | HLA-DQA1*01:02/DQB1*06:02 | DLSALTVLEAAELSK | 15 | 0.34 |
|  | HLA-DQA1*05:01/DQB1*03:01 | AGGAAPAAAAEEKTE | 15 | 0.35 |
|  | HLA-DRB1*09:01 | WGVSAAAPVAVAAAG | 15 | 0.35 |
|  | HLA-DQA1*05:01/DQB1*03:01 | LEEKWGVSAAAPVAV | 15 | 0.37 |
|  | HLA-DRB1*09:01 | LEEKWGVSAAAPVAV | 15 | 0.37 |
| omp16 | HLA-DQA1*05:01/DQB1*03:01 | DLGLGAGAATPGSSQ | 15 | 0.07 |
|  | HLA-DQA1*05:01/DQB1*03:01 | LGLGAGAATPGSSQD | 15 | 0.08 |
|  | HLA-DQA1*05:01/DQB1*03:01 | GDLGLGAGAATPGSS | 15 | 0.1 |
|  | HLA-DQA1*05:01/DQB1*03:01 | AGDLGLGAGAATPGS | 15 | 0.21 |
|  | HLA-DRB1*03:01 | DRIFFDLDSSLIRAD | 15 | 0.23 |
|  | HLA-DRB1*03:01 | GDRIFFDLDSSLIRA | 15 | 0.23 |
|  | HLA-DRB1*03:01 | RIFFDLDSSLIRADA | 15 | 0.24 |
|  | HLA-DRB5*01:01 | TREYNLALGQRRAAA | 15 | 0.25 |
|  | HLA-DRB5*01:01 | GTREYNLALGQRRAA | 15 | 0.26 |
|  | HLA-DQA1*05:01/DQB1*03:01 | NAGDLGLGAGAATPG | 15 | 0.27 |
|  | HLA-DRB5*01:01 | REYNLALGQRRAAAT | 15 | 0.28 |
|  | HLA-DQA1*05:01/DQB1*03:01 | GLGAGAATPGSSQDF | 15 | 0.31 |
|  | HLA-DRB3*02:02 | TRDFLASRGVPTNRM | 15 | 0.31 |
|  | HLA-DRB5*01:01 | RGTREYNLALGQRRA | 15 | 0.37 |
|  | HLA-DQA1*05:01/DQB1*03:01 | NNAGDLGLGAGAATP | 15 | 0.39 |
|  | HLA-DRB1*03:01 | VGDRIFFDLDSSLIR | 15 | 0.39 |
|  | HLA-DRB1*15:01 | ARSPIAIALFMSLAV | 15 | 0.44 |
|  | HLA-DRB1*15:01 | IAIALFMSLAVAGCA | 15 | 0.44 |
|  | HLA-DPA1*02:01/DPB1*14:01 | IQSIARSPIAIALFM | 15 | 0.44 |
|  | HLA-DRB1*15:01 | PIAIALFMSLAVAGC | 15 | 0.44 |
|  | HLA-DRB3*02:02 | RDFLASRGVPTNRMR | 15 | 0.44 |
|  | HLA-DRB1*15:01 | RSPIAIALFMSLAVA | 15 | 0.44 |
|  | HLA-DRB1*15:01 | SPIAIALFMSLAVAG | 15 | 0.44 |
|  | HLA-DRB3*02:02 | ATRDFLASRGVPTNR | 15 | 0.49 |
|  | HLA-DRB3*02:02 | SSQDFTVNVGDRIFF | 15 | 0.49 |
| OMP19 | HLA-DRB1*01:01 | ASLLSLAAAGIVLAG | 15 | 0.1 |
|  | HLA-DRB1*01:01 | KASLLSLAAAGIVLA | 15 | 0.1 |
|  | HLA-DRB1*01:01 | SKASLLSLAAAGIVL | 15 | 0.1 |
|  | HLA-DRB1*01:01 | GISKASLLSLAAAGI | 15 | 0.16 |
|  | HLA-DRB1*01:01 | ISKASLLSLAAAGIV | 15 | 0.16 |
|  | HLA-DQA1*05:01/DQB1*03:01 | LLSLAAAGIVLAGCQ | 15 | 0.19 |
|  | HLA-DQA1*05:01/DQB1*03:01 | LSLAAAGIVLAGCQS | 15 | 0.19 |
|  | HLA-DQA1*05:01/DQB1*03:01 | SLLSLAAAGIVLAGC | 15 | 0.21 |
|  | HLA-DQA1*05:01/DQB1*03:01 | ASLLSLAAAGIVLAG | 15 | 0.27 |
|  | HLA-DQA1*05:01/DQB1*03:01 | SLAAAGIVLAGCQSS | 15 | 0.29 |
|  | HLA-DQA1*05:01/DQB1*03:01 | LAAAGIVLAGCQSSR | 15 | 0.45 |
|  | HLA-DQA1*01:02/DQB1*06:02 | LLSLAAAGIVLAGCQ | 15 | 0.49 |
|  | HLA-DQA1*01:02/DQB1*06:02 | LSLAAAGIVLAGCQS | 15 | 0.5 |
| OMP25 | HLA-DRB1*01:01 | MTFKNLLGASLVAVI | 15 | 0.01 |
|  | HLA-DRB1*08:02 | ASLVAVITSTSAYAA | 15 | 0.05 |
|  | HLA-DQA1*03:01/DQB1*03:02 | TSAYAADAIVAQEPA | 15 | 0.08 |
|  | HLA-DQA1*03:01/DQB1*03:02 | STSAYAADAIVAQEP | 15 | 0.1 |
|  | HLA-DRB1*08:02 | SLVAVITSTSAYAAD | 15 | 0.14 |
|  | HLA-DQA1*03:01/DQB1*03:02 | TSTSAYAADAIVAQE | 15 | 0.16 |
|  | HLA-DRB1*08:02 | GASLVAVITSTSAYA | 15 | 0.18 |
|  | HLA-DRB3*01:01 | LYTGYNFDTGNNFIL | 15 | 0.25 |
|  | HLA-DRB1*01:01 | TFKNLLGASLVAVIT | 15 | 0.25 |
|  | HLA-DRB3*01:01 | YTGYNFDTGNNFILG | 15 | 0.25 |
|  | HLA-DRB1*08:02 | LVAVITSTSAYAADA | 15 | 0.31 |
|  | HLA-DRB3*01:01 | TGYNFDTGNNFILGL | 15 | 0.31 |
|  | HLA-DRB3*01:01 | GLYTGYNFDTGNNFI | 15 | 0.32 |
|  | HLA-DRB1*13:02 | ADAIVAQEPAPIAIA | 15 | 0.33 |
|  | HLA-DRB1*13:02 | AADAIVAQEPAPIAI | 15 | 0.35 |
|  | HLA-DQA1*05:01/DQB1*03:01 | FMPYIAGGVAFGGIK | 15 | 0.39 |
|  | HLA-DQA1*05:01/DQB1*03:01 | MPYIAGGVAFGGIKN | 15 | 0.39 |
|  | HLA-DPA1*02:01/DPB1*14:01 | GETQLRWSGAVRARA | 15 | 0.41 |
|  | HLA-DPA1*02:01/DPB1*14:01 | TQLRWSGAVRARAGY | 15 | 0.41 |
|  | HLA-DQA1*04:01/DQB1*04:02 | TSAYAADAIVAQEPA | 15 | 0.41 |
|  | HLA-DRB1*13:02 | DAIVAQEPAPIAIAP | 15 | 0.42 |
|  | HLA-DPA1*02:01/DPB1*14:01 | ETQLRWSGAVRARAG | 15 | 0.43 |
|  | HLA-DRB1*13:02 | YAADAIVAQEPAPIA | 15 | 0.5 |

**Supplementary Table 3.** **CTL epitopes with immunogenicity > 0 and antigenicity > 1**

| antigen | allele | peptide | length | percentile  rank | immunogenicity  score | antigenicity score |
| --- | --- | --- | --- | --- | --- | --- |
| L7/L12 | HLA-A*30:01 | RALTGLGLK | 9 | 0.1 | 0.08014 | 1.0154 |
|  | HLA-A*03:01 | RALTGLGLK | 9 | 0.26 | 0.08014 | 1.0154 |
|  | HLA-A*11:01 | RALTGLGLK | 9 | 0.29 | 0.08014 | 1.0154 |
|  | HLA-B*40:01 | EEKTEFDVVL | 10 | 0.39 | 0.2604 | 1.0723 |
|  | HLA-A*03:01 | VLADGGANK | 9 | 0.12 | 0.12916 | 1.0951 |
|  | HLA-A*11:01 | VLADGGANK | 9 | 0.31 | 0.12916 | 1.0951 |
|  | HLA-A*02:06 | AQLEAAGAKV | 10 | 0.39 | 0.11227 | 1.4394 |
|  | HLA-B*40:01 | LEAAGAKVEL | 10 | 0.12 | 0.01351 | 1.7008 |
| OMP16 | HLA-B*35:01 | SPIAIALFM | 9 | 0.09 | 0.30804 | 1.0874 |
|  | HLA-B*53:01 | SPIAIALFM | 9 | 0.13 | 0.30804 | 1.0874 |
|  | HLA-B*07:02 | SPIAIALFM | 9 | 0.3 | 0.30804 | 1.0874 |
|  | HLA-B*51:01 | SPIAIALFM | 9 | 0.41 | 0.30804 | 1.0874 |
|  | HLA-A*68:02 | SSQDFTVNV | 9 | 0.09 | 0.16632 | 1.1458 |
|  | HLA-A*02:06 | SSQDFTVNV | 9 | 0.15 | 0.16632 | 1.1458 |
|  | HLA-A*30:01 | GTREYNLAL | 9 | 0.31 | 0.12136 | 1.4159 |
|  | HLA-A*68:01 | ITIEGHADER | 10 | 0.22 | 0.3225 | 1.6912 |
|  | HLA-A*01:01 | HADERGTREY | 10 | 0.04 | 0.33007 | 2.2087 |
|  | HLA-B*35:01 | HADERGTREY | 10 | 0.12 | 0.33007 | 2.2087 |
|  | HLA-B*44:02 | ADERGTREY | 9 | 0.47 | 0.2563 | 2.3368 |
| OMP19 | HLA-A*68:02 | LTPGAVAGV | 9 | 0.16 | 0.16028 | 1.0179 |
|  | HLA-A*02:03 | VLYDANGGTV | 10 | 0.29 | 0.1361 | 1.0840 |
|  | HLA-B*07:02 | APDLTPGAV | 9 | 0.2 | 0.07486 | 1.1313 |
|  | HLA-A*68:02 | DLTPGAVAGV | 10 | 0.32 | 0.16422 | 1.1461 |
|  | HLA-A*30:02 | ANGGTVASLY | 10 | 0.36 | 0.01383 | 1.2679 |
| OMP25 | HLA-A*68:01 | AATDNVLLR | 9 | 0.22 | 0.05164 | 1.0024 |
|  | HLA-A*11:01 | AATDNVLLR | 9 | 0.33 | 0.05164 | 1.0024 |
|  | HLA-A*33:01 | DNVLLRLEYR | 10 | 0.13 | 0.11374 | 1.0320 |
|  | HLA-A*26:01 | WTVGAGIEY | 9 | 0.05 | 0.28832 | 1.1050 |
|  | HLA-A*01:01 | WTVGAGIEY | 9 | 0.14 | 0.28832 | 1.1050 |
|  | HLA-B*35:01 | WTVGAGIEY | 9 | 0.17 | 0.28832 | 1.1050 |
|  | HLA-A*30:02 | VLLRLEYRY | 9 | 0.13 | 0.15905 | 1.2541 |
|  | HLA-B*35:01 | APIAIAPSF | 9 | 0.02 | 0.14298 | 1.3619 |
|  | HLA-B*53:01 | APIAIAPSF | 9 | 0.03 | 0.14298 | 1.3619 |
|  | HLA-B*07:02 | APIAIAPSF | 9 | 0.04 | 0.14298 | 1.3619 |
|  | HLA-B*51:01 | APIAIAPSF | 9 | 0.19 | 0.14298 | 1.3619 |
|  | HLA-A*68:01 | DIRLGVAYK | 9 | 0.48 | 0.10836 | 1.4018 |
|  | HLA-A*68:02 | FKTNDIRLGV | 10 | 0.19 | 0.21645 | 1.4482 |
|  | HLA-B*58:01 | PIAIAPSFSW | 10 | 0.29 | 0.02033 | 1.7431 |
|  | HLA-B*57:01 | PIAIAPSFSW | 10 | 0.3 | 0.02033 | 1.7431 |
|  | HLA-A*30:01 | KTNDIRLGV | 9 | 0.06 | 0.20898 | 1.7838 |
|  | HLA-A*02:06 | KTNDIRLGV | 9 | 0.18 | 0.20898 | 1.7838 |
|  | HLA-A*68:02 | KTNDIRLGV | 9 | 0.19 | 0.20898 | 1.7838 |
|  | HLA-A*32:01 | RTNGGTSEF | 9 | 0.02 | 0.02042 | 1.9499 |
|  | HLA-B*15:01 | RTNGGTSEF | 9 | 0.08 | 0.02042 | 1.9499 |
|  | HLA-A*30:02 | RTNGGTSEF | 9 | 0.14 | 0.02042 | 1.9499 |
|  | HLA-B*58:01 | RTNGGTSEF | 9 | 0.15 | 0.02042 | 1.9499 |
|  | HLA-B*57:01 | RTNGGTSEF | 9 | 0.34 | 0.02042 | 1.9499 |
|  | HLA-B*57:01 | QTTGETQLRW | 10 | 0.04 | 0.09384 | 2.0563 |
|  | HLA-B*58:01 | QTTGETQLRW | 10 | 0.04 | 0.09384 | 2.0563 |
|  | HLA-B*58:01 | TTGETQLRW | 9 | 0.01 | 0.06139 | 2.0812 |
|  | HLA-B*57:01 | TTGETQLRW | 9 | 0.03 | 0.06139 | 2.0812 |
|  | HLA-A*02:06 | FILGLDANV | 9 | 0.17 | 0.06982 | 2.1148 |
|  | HLA-A*02:01 | FILGLDANV | 9 | 0.28 | 0.06982 | 2.1148 |
|  | HLA-A*68:01 | QTTGETQLR | 9 | 0.07 | 0.0765 | 2.2398 |
|  | HLA-A*03:01 | RTNGGTSEFK | 10 | 0.05 | 0.09997 | 2.2491 |
|  | HLA-A*11:01 | RTNGGTSEFK | 10 | 0.07 | 0.09997 | 2.2491 |
|  | HLA-A*30:01 | RTNGGTSEFK | 10 | 0.11 | 0.09997 | 2.2491 |

**Supplementary Table 4. HTL epitopes with positive IFN-γ induction and an antigenicity score of >0**

| antigen | allele | peptide | length | rank | antigenicity score | INF-γ |
| --- | --- | --- | --- | --- | --- | --- |
| L7L12 | HLA-DQA1*05:01/DQB1*03:01 | AAAGGAAPAAAAEEK | 15 | 0.01 | 1.1764 | 0.99845044 |
|  | HLA-DQA1*05:01/DQB1*03:01 | AAAPVAVAAAGGAAP | 15 | 0.03 | 0.8218 | 0.41718742 |
|  | HLA-DQA1*05:01/DQB1*03:01 | AAGGAAPAAAAEEKT | 15 | 0.02 | 1.2062 | 0.67010683 |
|  | HLA-DQA1*05:01/DQB1*03:01 | AAPVAVAAAGGAAPA | 15 | 0.02 | 0.8452 | 0.11843766 |
|  | HLA-DQA1*05:01/DQB1*03:01 | AGGAAPAAAAEEKTE | 15 | 0.35 | 1.2709 | 0.4695206 |
|  | HLA-DQA1*05:01/DQB1*03:01 | APVAVAAAGGAAPAA | 15 | 0.01 | 0.8299 | 0.11843766 |
|  | HLA-DQA1*05:01/DQB1*03:01 | AVAAAGGAAPAAAAE | 15 | 0.01 | 0.9646 | 0.78932102 |
|  | HLA-DQA1*05:01/DQB1*03:01 | PVAVAAAGGAAPAAA | 15 | 0.01 | 0.8975 | 0.57531119 |
|  | HLA-DQA1*05:01/DQB1*03:01 | SAAAPVAVAAAGGAA | 15 | 0.15 | 0.9471 | 0.40277536 |
|  | HLA-DQA1*05:01/DQB1*03:01 | VAAAGGAAPAAAAEE | 15 | 0.01 | 0.9596 | 0.97868375 |
|  | HLA-DQA1*05:01/DQB1*03:01 | VAVAAAGGAAPAAAA | 15 | 0.01 | 0.8829 | 0.89056137 |
| OMP16 | HLA-DRB1*03:01 | DRIFFDLDSSLIRAD | 15 | 0.23 | 0.4029 | 0.080503905 |
|  | HLA-DRB5*01:01 | TREYNLALGQRRAAA | 15 | 0.25 | 0.7344 | 0.14599306 |
|  | HLA-DQA1*05:01/DQB1*03:01 | NAGDLGLGAGAATPG | 15 | 0.27 | 1.2041 | 0.1272639 |
|  | HLA-DRB5*01:01 | REYNLALGQRRAAAT | 15 | 0.28 | 0.6696 | 0.58595332 |
|  | HLA-DRB3*02:02 | TRDFLASRGVPTNRM | 15 | 0.31 | 0.1695 | 0.15854351 |
|  | HLA-DRB5*01:01 | RGTREYNLALGQRRA | 15 | 0.37 | 0.8472 | 0.1543397 |
|  | HLA-DRB3*02:02 | RDFLASRGVPTNRMR | 15 | 0.44 | 0.1687 | 0.20784873 |
|  | HLA-DRB3*02:02 | ATRDFLASRGVPTNR | 15 | 0.49 | 0.1774 | 0.279096 |
| OMP25 | HLA-DQA1*03:01/DQB1*03:02 | TSAYAADAIVAQEPA | 15 | 0.08 | 0.5802 | 0.27367041 |
|  | HLA-DQA1*03:01/DQB1*03:02 | TSTSAYAADAIVAQE | 15 | 0.16 | 0.6488 | 0.48824974 |
|  | HLA-DRB3*01:01 | YTGYNFDTGNNFILG | 15 | 0.25 | 0.4107 | 0.2823727 |
|  | HLA-DRB3*01:01 | TGYNFDTGNNFILGL | 15 | 0.31 | 0.6263 | 0.43434236 |
|  | HLA-DPA1*02:01/DPB1*14:01 | GETQLRWSGAVRARA | 15 | 0.41 | 0.8089 | 0.64072688 |
|  | HLA-DPA1*02:01/DPB1*14:01 | TQLRWSGAVRARAGY | 15 | 0.41 | 0.7484 | 0.88285166 |
|  | HLA-DQA1*04:01/DQB1*04:02 | TSAYAADAIVAQEPA | 15 | 0.41 | 0.5802 | 0.27367041 |
|  | HLA-DPA1*02:01/DPB1*14:01 | ETQLRWSGAVRARAG | 15 | 0.43 | 0.8818 | 0.58427055 |
|  | HLA-DRB1*13:02 | YAADAIVAQEPAPIA | 15 | 0.5 | 0.4301 | 0.020304485 |

**Supplementary Table 5.** **Predicted candidate B-cell epitopes**

| antigen | peptide | score |
| --- | --- | --- |
| L7L12 | AAAAEEKTEFDVVLAD | 0.87 |
|  | EAEKIKAQLEAAGAKV | 0.74 |
|  | KDLVEGAPKAVKEGAS | 0.73 |
|  | AVKEGASKDEAEKIKA | 0.7 |
|  | DVVLADGGANKINVIK | 0.67 |
|  | AAELSKLLEEKWGVSA | 0.67 |
|  | GVSAAAPVAVAAAGGA | 0.66 |
|  | TGLGLKEAKDLVEGAP | 0.64 |
|  | GGANKINVIKEVRALT | 0.62 |
|  | LLEEKWGVSAAAPVAV | 0.62 |
|  | EDLSALTVLEAAELSK | 0.6 |
| OMP16 | LGLGAGAATPGSSQDF | 0.91 |
|  | EGHADERGTREYNLAL | 0.9 |
|  | MRTISYGNERPVAVCD | 0.88 |
|  | AGCASKKNLPNNAGDL | 0.87 |
|  | AATPGSSQDFTVNVGD | 0.86 |
|  | QYSITIEGHADERGTR | 0.82 |
|  | ASRGVPTNRMRTISYG | 0.82 |
|  | TLSKQAQWLQRYPQYS | 0.8 |
|  | LGQRRAAATRDFLASR | 0.79 |
|  | RRIQSIARSPIAIALF | 0.78 |
|  | VCDADTCWSQNRRAVT | 0.78 |
|  | TVNVGDRIFFDLDSSL | 0.77 |
|  | TREYNLALGQRRAAAT | 0.77 |
|  | ARSPIAIALFMSLAVA | 0.7 |
|  | FDLDSSLIRADAQQTL | 0.64 |
|  | CWSQNRRAVTVLNGAG | 0.61 |
|  | LIRADAQQTLSKQAQW | 0.6 |
| OMP19 | IATPQTKYGQGYRAGP | 0.93 |
|  | TQVASLPPASAPDLTP | 0.9 |
|  | ASAPDLTPGAVAGVWN | 0.88 |
|  | AGTVQKGNLDSPTQFP | 0.87 |
|  | NLDNVSPPPPPAPVNA | 0.84 |
|  | QGRFDGQTTGGQAVTL | 0.84 |
|  | VWNASLGGQSCKIATP | 0.82 |
|  | AAGIVLAGCQSSRLGN | 0.82 |
|  | GYRAGPLRCPGELANL | 0.82 |
|  | AGCQSSRLGNLDNVSP | 0.81 |
|  | TDMSAQSGTQVASLPP | 0.8 |
|  | ASLYSSGQGRFDGQTT | 0.79 |
|  | DSPTQFPNAPSTDMSA | 0.77 |
|  | GELANLASWAVNGKQL | 0.72 |
|  | AVNGKQLVLYDANGGT | 0.71 |
|  | MGISKASLLSLAAAGI | 0.69 |
|  | LVLYDANGGTVASLYS | 0.67 |
|  | PPPPPAPVNAVPAGTV | 0.65 |
| OMP25 | GWTVGAGIEYAATDNV | 0.94 |
|  | FGGQVGYGWGRAKLEN | 0.92 |
|  | APIAIAPSFSWAGAYF | 0.91 |
|  | IGGLYTGYNFDTGNNF | 0.9 |
|  | YGWGRAKLENRTNGGT | 0.9 |
|  | MPYIAGGVAFGGIKNS | 0.9 |
|  | VITSTSAYAADAIVAQ | 0.9 |
|  | EYRYTDYGKKNFGLND | 0.89 |
|  | GTSEFKPNGFIGGLYT | 0.88 |
|  | LKKSRDFITSGNPVQT | 0.83 |
|  | FGLNDLDTRGSFKTND | 0.82 |
|  | FGGIKNSLRIGGEESS | 0.82 |
|  | SLRIGGEESSKSKTQT | 0.8 |
|  | QLRWSGAVRARAGYAI | 0.8 |
|  | FILGLDANVDYNNLKK | 0.78 |
|  | ARAGYAIDRFMPYIAG | 0.77 |
|  | DAIVAQEPAPIAIAPS | 0.71 |
|  | VQTTGETQLRWSGAVR | 0.7 |
|  | PSFSWAGAYFGGQVGY | 0.68 |
|  | GYNFDTGNNFILGLDA | 0.62 |
|  | TRGSFKTNDIRLGVAY | 0.62 |
|  | KNLLGASLVAVITSTS | 0.59 |

**Supplementary Table 6. Discontinuous B-cell epitopes in multi-epitope vaccines predicted by the ElliPro server**

| No | Residues | Number of residues | score |
| --- | --- | --- | --- |
| 1 | A:I30, A:G31, A:K32 | 3 | 0.854 |
| 2 | A:A275, A:E278, A:K279, A:T280, A:G281, A:P282, A:G283, A:P284, A:G285, A:A286, A:G287, A:G288, A:A289, A:A290, A:P291, A:A292, A:A293, A:A294, A:A295, A:E296, A:E297, A:K298, A:T299, A:E300, A:G301, A:P302, A:G303, A:P304, A:G305, A:L306, A:G307, A:L308, A:G309, A:A310, A:G311, A:A312, A:A313, A:T314, A:P315, A:G316, A:S317, A:S318, A:Q319, A:F321 | 44 | 0.817 |
| 3 | A:V501, A:Y502, A:T503, A:I504, A:P505, A:P506, A:P507, A:K508, A:E509, A:Q510, A:M511, A:A512, A:K513, A:D514, A:K515, A:V516, A:S517, A:L518, A:T519, A:C520, A:E533, A:W534, A:Q535, A:W536, A:N537, A:G538, A:Q539, A:P540, A:A541, A:E542, A:N543, A:Y544, A:S561, A:K562, A:L563, A:N564, A:V565, A:Q566, A:K567, A:S568, A:N569, A:W570, A:E571, A:A572, A:G573, A:N574, A:T575, A:F576, A:T577, A:C578, A:S579, A:L585, A:H586, A:N587, A:H589, A:T590, A:E591, A:K592, A:S593, A:L594, A:S595, A:H596, A:S597, A:P598, A:G599, A:K600 | 66 | 0.791 |
| 4 | A:G1, A:I2, A:I3, A:N4, A:T5, A:L6, A:Q7, A:K8, A:Y10, A:C11, A:R12, A:G16, A:R17, A:C18, A:A19, A:V20, A:L21, A:S22, A:C23, A:L24, A:P25, A:K26, A:E27, A:E28, A:Q29, A:C33, A:S34, A:T35, A:R36, A:G37, A:R38, A:C40, A:C41, A:K44, A:A55, A:A56, A:T58, A:L59, A:K60, A:A61, A:A62, A:A63, A:G64, A:P65, A:G66, A:P67, A:G68, A:H69, A:A70, A:D71, A:E72, A:R73, A:G74, A:T75, A:R76, A:E77, A:Y78, A:G79, A:P80, A:G81, A:P82, A:G83, A:A84, A:D85, A:E86, A:R87, A:G88, A:T89, A:E91, A:Y92, A:G93, A:P94, A:G95, A:P96, A:G97, A:L98, A:T99, A:P100, A:G101, A:A102, A:V103, A:A104, A:G105, A:V106, A:G107, A:P108, A:G109, A:P110, A:G111, A:D112, A:G120, A:V121, A:G122, A:P123, A:G124, A:P125, A:G126, A:F127, A:K128, A:T129, A:N130, A:D131, A:I132, A:R133, A:A176, A:G177, A:A178, A:K179 | 108 | 0.725 |
| 5 | A:L382, A:D384, A:K385, A:V387, A:P388, A:E389, A:V390, A:S391, A:D418, A:I419, A:S420, A:K421, A:D422, A:D423, A:P424, A:E425, A:V426, A:Q427, A:F428, A:S429, A:D434, A:V435, A:E436, A:V437, A:H438, A:T439, A:A440, A:Q441, A:T442, A:Q443, A:P444, A:R445, A:E446, A:E447, A:Q448, A:F449, A:N450, A:S451, A:T452, A:F453, A:R454, A:S455, A:V456, A:S457, A:E458, A:P460, A:I461, A:V476, A:N477, A:S478, A:A479, A:A480, A:F481, A:P482, A:A483, A:P484 | 56 | 0.652 |
| 6 | A:A238, A:R239, A:A240, A:G241, A:P242, A:G243, A:P244, A:G245 | 8 | 0.604 |
| 7 | A:I327, A:A328, A:T329, A:P330, A:Q331, A:T332, A:K333, A:Y334, A:G335, A:Q336, A:G337, A:Y338, A:R339 | 13 | 0.553 |
| 8 | A:T405, A:I406, A:T407, A:L408, A:T409, A:P410 | 6 | 0.53 |
| 9 | A:G364, A:P365, A:G366, A:P367, A:G368, A:A369, A:A370 | 7 | 0.524 |

**Supplementary Table 7. Consecutive B-cell epitopes in multi-epitope vaccines predicted by the ElliPro server**

| No | Peptide | Number of residues | score |
| --- | --- | --- | --- |
| 1 | EKTGPGPGAGGAAPAAAAEEKTEGPGPGLGLGAGAATPGSSQ | 42 | 0.832 |
| 2 | SKLNVQKSNWEAGNTFTCS | 19 | 0.816 |
| 3 | VYTIPPPKEQMAKDKVSLTC | 20 | 0.81 |
| 4 | AAWTLKAAAGPGPGHADERGTREYGPGPGADERGTREYGPGPGLTPGAVAGVGPGPGD | 58 | 0.76 |
| 5 | EWQWNGQPAENYK | 13 | 0.753 |
| 6 | DISKDDPEVQFS | 12 | 0.738 |
| 7 | LHNHHTEKSLSHSPGK | 16 | 0.73 |
| 8 | GVGPGPGFKTNDIRL | 15 | 0.71 |
| 9 | GRCAVLSCLPKEEQIGKCSTRGRKCCRRK | 29 | 0.695 |
| 10 | DVEVHTAQTQPREEQFNSTFRSVSELPIM | 29 | 0.65 |
| 11 | VNSAAFPAP | 9 | 0.627 |
| 12 | GIINTLQKYYCR | 12 | 0.613 |
| 13 | ARAGPGPG | 8 | 0.604 |
| 14 | IATPQTKYGQG | 11 | 0.582 |
| 15 | DKKVPEVS | 8 | 0.569 |
| 16 | PGPGAA | 6 | 0.546 |
| 17 | TITLTP | 6 | 0.53 |
| 18 | AGAK | 4 | 0.512 |

**Supplementary Table 8. Hydrogen bonding between MEV-Fc-TLR2 complexes**

| no | Structure 1 | dist | Structure 2 |
| --- | --- | --- | --- |
| 1 | A:HIS 358[ NE2] | 3.89 | B:ARG 239[ O ] |
| 2 | A:ASN 379[HD22] | 2.15 | B:GLY 368[ O ] |
| 3 | A:TYR 326[ HH ] | 1.83 | B:LYS 375[ O ] |
| 4 | A:ARG 321[HH12] | 1.89 | B:GLU 377[ OE1] |
| 5 | A:ARG 321[HH11] | 2.24 | B:GLU 377[ OE2] |
| 6 | A:ARG 321[HH11] | 2.1 | B:PHE 378[ O ] |
| 7 | A:ARG 321[HH21] | 1.85 | B:PHE 378[ O ] |
| 8 | A:ARG 321[HH22] | 1.94 | B:ASP 379[ OD1] |
| 9 | A:LYS 347[ HZ1] | 1.74 | B:GLU 447[ O ] |
| 10 | A:LYS 347[ HZ2] | 1.66 | B:GLN 448[ O ] |
| 11 | A:PHE 325[ O ] | 1.67 | B:LYS 375[ HZ2] |

**Supplementary Table 9.** **Hydrogen bonding between MEV-Fc-TLR4 complexes**

| no | Structure 1 | dist | Structure 2 |
| --- | --- | --- | --- |
| 1 | A:ARG 67[HH11] | 2.05 | B:ARG 142[ O ] |
| 2 | A:ARG 67[HH12] | 1.91 | B:THR 143[ OG1] |
| 3 | A:LYS 47[ HZ3] | 1.75 | B:ASN 144[ O ] |
| 4 | A:LYS 47[ HZ1] | 1.67 | B:ASN 144[ OD1] |
| 5 | A:LYS 47[ HZ2] | 1.65 | B:GLY 145[ O ] |
| 6 | A:GLN 91[HE21] | 1.85 | B:ASN 164[ O ] |
| 7 | A:GLN 91[HE22] | 2.2 | B:LYS 165[ O ] |
| 8 | A:GLN 578[HE21] | 2.28 | B:PRO 302[ O ] |
| 9 | A:ARG 87[HH11] | 1.87 | B:PRO 342[ O ] |
| 10 | A:ARG 87[HH21] | 1.97 | B:PRO 342[ O ] |
| 11 | A:GLN 39[HE22] | 1.99 | B:GLY 348[ O ] |
| 12 | A:GLU 31[ H ] | 2.05 | B:VAL 351[ O ] |
| 13 | A:ASN 35[HD22] | 2.17 | B:VAL 390[ O ] |
| 14 | A:PHE 54[ H ] | 2 | B:PRO 484[ O ] |
| 15 | A:HIS 68[ O ] | 1.98 | B:GLY 146[ H ] |
| 16 | A:GLU 89[ OE1] | 2.01 | B:ASN 164[HD22] |
| 17 | A:GLU 89[ OE1] | 1.99 | B:ASN 186[HD22] |
| 18 | A:GLU 42[ OE1] | 2.01 | B:GLY 226[ H ] |
| 19 | A:GLU 27[ O ] | 1.82 | B:ARG 255[HH12] |
| 20 | A:GLU 27[ O ] | 1.94 | B:ARG 255[HH22] |
| 21 | A:GLU 42[ O ] | 2.45 | B:ARG 257[HH22] |
| 22 | A:GLU 42[ OE2] | 1.85 | B:ARG 257[HH22] |
| 23 | A:ASP 238[ OD2] | 1.96 | B:GLN 331[HE22] |
| 24 | A:LEU 212[ O ] | 2.16 | B:ARG 339[ HE ] |
| 25 | A:GLU 31[ OE2] | 1.91 | B:ALA 383[ H ] |
| 26 | A:ASN 51[ O ] | 1.76 | B:LYS 487[ HZ3] |
| 27 | A:ASN 51[ OD1] | 1.71 | B:LYS 487[ HZ2] |
